# RBFOX2 recognizes *N*^6^-methyladenosine to suppress transcription and block myeloid leukaemia differentiation

**DOI:** 10.1038/s41556-023-01213-w

**Published:** 2023-08-28

**Authors:** Xiaoyang Dou, Yu Xiao, Chao Shen, Kitty Wang, Tong Wu, Chang Liu, Yini Li, Xianbin Yu, Jun Liu, Qing Dai, Kinga Pajdzik, Chang Ye, Ruiqi Ge, Boyang Gao, Jianhua Yu, Shuying Sun, Mengjie Chen, Jianjun Chen, Chuan He

**Affiliations:** 1 Department of Chemistry, Department of Biochemistry and Molecular Biology, and Institute for Biophysical Dynamics, The University of Chicago, Chicago, IL USA; 2 Howard Hughes Medical Institute, Chicago, IL USA; 3 Department of Systems Biology, Beckman Research Institute of City of Hope, Monrovia, CA USA; 4 City of Hope Comprehensive Cancer Center, City of Hope, Duarte, CA USA; 5 Gehr Family Center for Leukemia Research, City of Hope, Duarte, CA USA; 6 Department of Physiology and Brain Science Institute, Johns Hopkins University School of Medicine, Baltimore, MD USA; 7 Department of Hematology and Hematopoietic Cell Transplantation, City of Hope National Medical Center, Duarte, CA USA; 8 Hematologic Malignancies Research Institute, City of Hope National Medical Center, Duarte, CA USA; 9 Section of Genetic Medicine, Department of Medicine, University of Chicago, Chicago, IL USA; 10 Department of Human Genetics, University of Chicago, Chicago, IL USA; 11 Present Address: State Key Laboratory of Protein and Plant Gene Research, School of Life Sciences, Peking-Tsinghua Center for Life Sciences, Peking University, Beijing, China

**Keywords:** Gene silencing, Cancer models, Transcriptional regulatory elements

## Abstract

*N*^6^-methyladenosine (m^6^A) methylation can be deposited on chromatin-associated RNAs (caRNAs) by the RNA methyltransferase complex (MTC) to regulate chromatin state and transcription. However, the mechanism by which MTC is recruited to distinct genomic loci remains elusive. Here we identify RBFOX2, a well-studied RNA-binding protein, as a chromatin factor that preferentially recognizes m^6^A on caRNAs. RBFOX2 can recruit RBM15, an MTC component, to facilitate methylation of promoter-associated RNAs. RBM15 also physically interacts with YTHDC1 and recruits polycomb repressive complex 2 (PRC2) to the RBFOX2-bound loci for chromatin silencing and transcription suppression. Furthermore, we found that this RBFOX2/m^6^A/RBM15/YTHDC1/PRC2 axis plays a critical role in myeloid leukaemia. Downregulation of RBFOX2 notably inhibits survival/proliferation of acute myeloid leukaemia cells and promotes their myeloid differentiation. RBFOX2 is also required for self-renewal of leukaemia stem/initiation cells and acute myeloid leukaemia maintenance. Our study presents a pathway of m^6^A MTC recruitment and m^6^A deposition on caRNAs, resulting in locus-selective chromatin regulation, which has potential therapeutic implications in leukaemia.

## Abstract (web summary)

Dou, Xiao, Shen, Wang et al. show that RBFOX2 recognizes m^6^A on chromatin-associated RNAs and recruits RBM15, YTHDC1 and PRC2 to facilitate transcription suppression. Inhibition of the axis exerts anti-leukaemic effects.

## Main

*N*^6^-methyladenosine (m^6^A) is the most prevalent internal modification of messenger RNAs in mammalian cells and also exists in non-coding RNAs such as chromatin-associated regulatory RNAs (carRNAs)^1,2^. This modification is installed by the m^6^A methyltransferase complex (MTC), consisting of METTL3/14 (refs. ^3–6^), WTAP^7^, RBM15/15B^8^, ZC3H13 (ref. ^9^) and VIRMA (KIAA1429) (ref. ^10^), and can be removed by the demethylases FTO^11^ and ALKBH5 (ref. ^12^). The functional effects of m^6^A on mRNA metabolisms and translation are mediated through m^6^A reader proteins, such as the YTH domain-containing proteins (YTHDC1/2 (refs. ^13–15^) and YTHDF1/2/3 (refs. ^16–18^)). The list of reader proteins that can bind preferentially to the m^6^A-modified transcripts through direct or indirect mechanisms, including IGF2BP1/2/3 (ref. ^19^), HNRNPA2B1 (ref. ^20^), HNRNPC^21^ and HuR^22^, continues to expand.

While the critical roles of m^6^A in post-transcriptional mRNA decay and translation regulation have been well established, recent studies have also revealed that the METTL3/METTL14 complex can mediate m^6^A methylation of non-coding chromatin-associated RNAs (caRNAs) to modulate chromatin state and regulate transcription^1,23–27^; however, how MTC is recruited to different chromatin loci and how it achieves locus-selective regulation are largely unknown. In addition, YTHDC1 is the only m^6^A reader protein identified that interfaces m^6^A with chromatin regulation^1,25,26,28^. However, how YTHDC1 is recruited to engage the m^6^A-dependent, locus-selective regulation is still unclear.

In this Article, we show a transcriptional regulatory axis involving MTC, YTHDC1 and an RNA-binding protein (RBP) RBFOX2: RBFOX2 recruits RBM15, an MTC component, to methylate promoter-associated RNAs (paRNAs). RBM15 further interacts with YTHDC1 to recruit PRC2 to the RBFOX2-bound loci for transcription suppression. The RBFOX2/m^6^A/RBM15/YTHDC1/PRC2 axis plays an important role in myeloid leukaemia, with downregulation of RBFOX2 notably inhibiting acute myeloid leukaemia (AML) cell survival/proliferation and promoting myeloid differentiation of AML cells. Therefore, RBFOX2 is identified as a chromatin factor that facilitates m^6^A deposition on caRNAs during locus-specific chromatin regulation with therapeutic implications in leukaemia.

## Results

### Protein candidates that may bind caRNA m^6^A

**Fig. 1:**
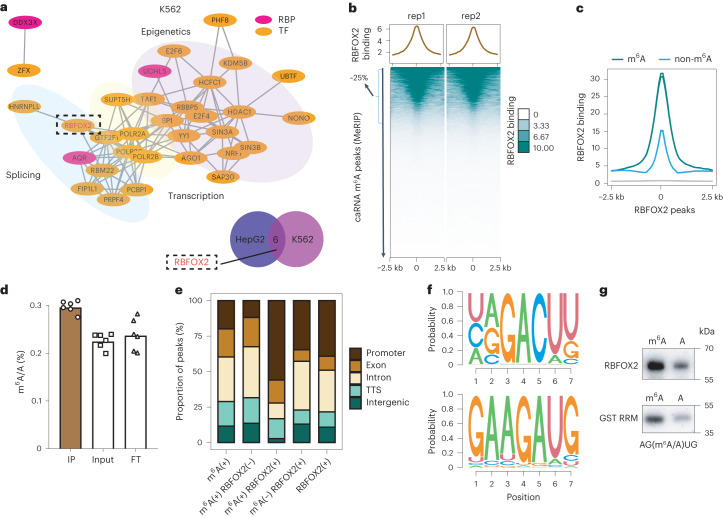
RBFOX2 recognizes m^6^A on paRNA. **a**, Top: a protein–protein interaction network with the top-ranked (*n* = 50) m^6^A-associated proteins on caRNAs in K562 cells. Bottom: Venn diagram of top-ranked (*n* = 20) m^6^A-associated proteins on caRNAs between K562 and HepG2 cells. The highlighted protein (dotted circle) is shared between K562 and HepG2 cells. **b**, Average profile (top) and heat map (bottom) showing RBFOX2 binding intensity at m^6^A peak centres and the flanking 2.5 kb regions in K562 cells. rep, replicate. **c**, Average profile of RBFOX2 binding intensity at RBFOX2 peak centres and the flanking 2.5 kb regions in K562 cells. RBFOX2 peaks^29,30^ were categorized into two groups according to whether they overlapped with m^6^A (m^6^A) or not (non-m^6^A). **d**, LC–MS/MS showing m^6^A enrichment in RBFOX2-bound RNA while depleted in the FT portion (*n* = 6, three technical replicates over two biological replicates). Data are represented as mean values ± standard deviation. Two-sided *P* value was calculated by Student’s *t*-test. **e**, Distribution of m^6^A or RBFOX2 peaks^29,30^ at distinct genomic regions including promoter, exonic, intronic, transcription termination sites (TTS) and intergenic regions annotated by HOMER^48^ in K562 cells. ‘(+)’ sign represents regions harbouring m^6^A (+) or bound by RBFOX2 (+), while the sign ‘(−)’ indicates the absence of m^6^A (−) or RBFOX2 (−). **f**, Top consensus sequences on RBFOX2-bound sites that were marked with m^6^A (caRNA m^6^A-SAC-seq) using HOMER^48^ motif discovery algorithm. **g**, Oligo pulldown assay showing RBFOX2 (top) and RRM domain of RBFOX2 (bottom) bound an m^6^A-containing RNA probe with higher affinity than the unmethylated control. Source data

**Extended Data Fig. 1:**
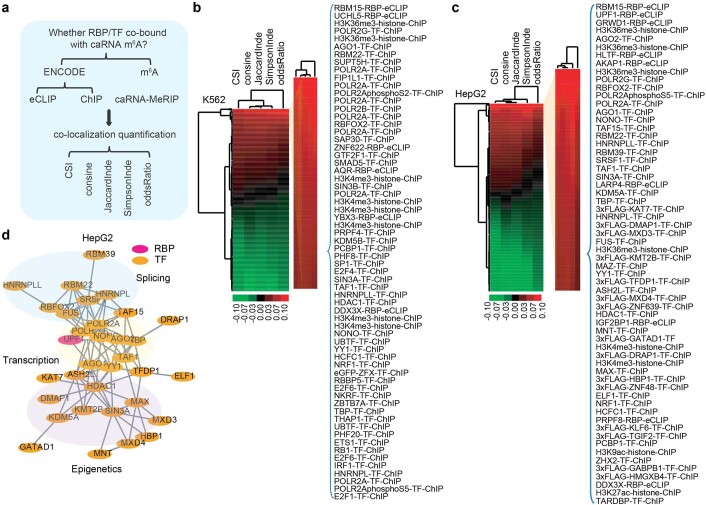
Identification of proteins associated with m^6^A on chromatin-associated RNAs. **a**, A diagram showing the analysis pipeline for identification of proteins associated with m^6^A on chromatin-associated RNAs. **b-c**, Heatmap showing the association score of binding profile between proteins (ENCODE project) and m^6^A of chromatin-associated RNAs (this study) in K562 (***b***) and HepG2 (***c***) cells. **d**, A protein-protein interaction (PPI) network with the top-ranked (*n* = 50) m^6^A associated proteins on chromatin-associated RNAs in HepG2 cells. The depicted genome-wide data represent an integration of all samples, including two biologically independent replicates.

To identify potential transcription factors (TFs) and other chromatin factors involved in MTC recruitment to chromatin for the m^6^A-dependent regulation, we first performed an integrative analysis using a large collection of high-throughput, genome- and transcriptome-wide protein-binding data from the ENCODE^29,30^ project. We used TF/histone modification chromatin immunoprecipitation followed by sequencing (ChIP–seq) datasets for K562 (*n* = 540) and HepG2 (*n* = 313) cells, as well as RBP enhanced crosslinking and immunoprecipitation followed by sequencing (eCLIP-seq) datasets for K562 (*n* = 120) and HepG2 (*n* = 103) cells. Specifically, we associated the binding profiles with m^6^A peaks on caRNAs and ranked their co-localization using different indexes (Extended Data Fig. 1a–c). The top-ranked m^6^A-associated proteins include RBM22 and POLR2A, which are known to be involved in splicing^31^ and transcription regulation^32^ (Fig. 1a and Extended Data Fig. 1d), consistent with the well-studied functions of m^6^A. Our analysis also revealed strong co-localization of m^6^A with chromatin regulators, congruous with recent discoveries of the m^6^A regulation of chromatin state^1,23,25–27,33^. Among the six proteins identified in both K562 and HepG2 cells (Fig. 1a), RBFOX2 drew our interest as it was recently reported to bind nascent RNA^34^, suggesting its potential role in regulating the m^6^A-modified caRNAs.

### RBFOX2 functions as an m^6^A-binding protein

**Extended Data Fig. 2:**
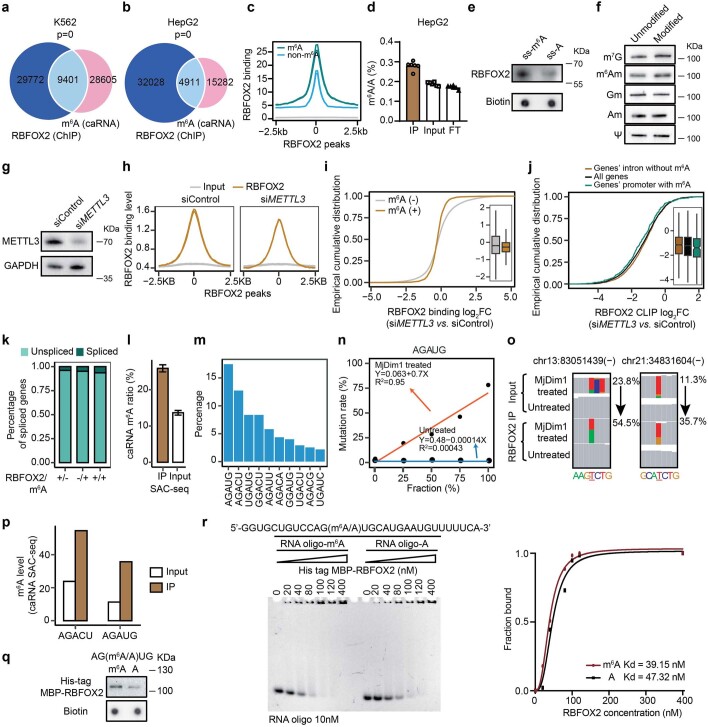
RBFOX2 binds m^6^A-marked promoter-associated RNAs. **a-b**, Peaks overlap between RBFOX2 (ChIP-seq, ENCODE project) and m^6^A (MeRIP-seq) of caRNAs in K562 (***a***) and HepG2 (***b***) cells. Two-sided *P* values by Fisher’s exact test. **c**, Average profile of RBFOX2 binding intensity at RBFOX2 peaks (m^6^A *versus* non-m^6^A, HepG2). **d**, LC-MS/MS showing m^6^A enrichment in RBFOX2-bound RNA while depleted in the flow-through (FT) portion (HepG2) (*n* = 6, three technical replicates over two biologically replicates). Mean values +/− SD. Two-sided *P* value by Student’s t-test. **e-f**, Western blot showing RBFOX2 pulled down by RNA probes containing m^6^A (**e**, 5′CGUGG(A/m^6^A)CUGGCUU-3′) or m^7^G/m^6^A_m_/G_m_/A_m_/Ψ (**f**), as well as their corresponding unmethylated control probes. **g**, METTL3 expression in K562 cells. **h**, Average profile of RBFOX2 binding at RBFOX2 peaks in K562 cells. **i**, RBFOX2 binding changes at RBFOX2 binding sites (*N* = 2082 m^6^A methylated *versus N* = 20846 unmethylated ones) upon *METTL3* knockdown in K562 cells. **j**, RBFOX2 CLIP binding log_2_FC upon *METTL3* KD in K562 cells. The RBFOX2 bindings on chromatin (*N*) were separated into three groups: those with m^6^A at their promoter regions (RBFOX2 may function as an m^6^A-binding protein, *N* = 272), those without m^6^A at their intronic regions (RBFOX2 may function as a splicing regulator, *N* = 1668), and all detected genes as background (*N* = 3673). **k**, The percentage of alternatively spliced genes by *RBFOX2* knockdown in K562 cells. **l**, m^6^A level in RBFOX2-bound caRNAs (IP) and the corresponding input caRNAs (Input) in K562 cells (*N* = 275 m^6^A sites, m^6^A-SAC-seq). Mean values +/− SEM. **m**, The percentage of m^6^A sites in different sequence contexts (motifs) in K562 cells (m^6^A-SAC-seq). **n**, Calibration curve for m^6^A within the AGAUG motif is generated by linear regression. **o-p**, The IGV visualization (**o**) and quantification (**p**) of the mutation frequency of identified m^6^A sites (m^6^A-SAC-seq). **q**, Oligo pulldown assay showing full length RBFOX2 bound to an m^6^A-containing RNA probe with higher affinity than the unmethylated control. **r**, EMSA assay measuring the dissociation constant (Kd, nM) of His-tagged MBP-RBFOX2 protein with methylated or unmethylated RNA probes. Source data

After overlapping RBFOX2 genomic binding sites with m^6^A peaks on caRNAs, we found that ~25% of caRNA m^6^A sites co-localize with chromatin binding sites of RBFOX2 in both K562 and HepG2 cells (Fig. 1b and Extended Data Fig. 2a,b). Furthermore, RBFOX2 showed a higher binding intensity on m^6^A-marked regions compared with non-marked ones, suggesting that either RBFOX2 recognizes m^6^A or m^6^A facilitates the binding of RBFOX2 to chromatin (Fig. 1c and Extended Data Fig. 2c). Next, we pulled down RBFOX2, isolated the bound RNAs and performed liquid chromatography–tandem mass spectrometry (LC–MS/MS) to measure the m^6^A/A ratio. The RBFOX2-bound RNAs showed higher a m^6^A/A ratio than controls, indicating preferential binding of RBFOX2 to m^6^A-containing RNA (Fig. 1d and Extended Data Fig. 2d). To validate this observation, we employed single-stranded bait RNA probes with or without m^6^A to pull down RBPs. Consistently, western blot assay showed that RBFOX2 bound to m^6^A-methylated bait (ss-m^6^A) with a higher affinity compared with unmethylated controls (ss-A) (Extended Data Fig. 2e). RBFOX2 pulldown using RNA probes containing less abundant RNA modifications revealed a slight preference of RBFOX2 for m^7^G and m^6^A_m_ (Extended Data Fig. 2f). The slight preference for m^6^A_m_ is expected as it is the same base modification as m^6^A but in much lower abundance. The slight preference for m^7^G warrants future investigations. Besides, we did find that *METTL3* knockdown (KD) resulted in a global decrease in RBFOX2 binding on chromatin, particularly at regions that are both RBFOX2 bound and m^6^A marked (Extended Data Fig. 2g–j), further supporting the important role of m^6^A in the chromatin binding of RBFOX2. Altogether, these results support RBFOX2 as an RBP that preferentially recognizes m^6^A-modified RNA.

Given that RBFOX2 is a well-known splicing regulator that binds introns adjacent to alternatively spliced exons^35^, we tested whether or not the regulation of RBFOX2 on m^6^A is dependent on its splicing role. To this end, we categorized RBFOX2 genomic binding sites into m^6^A-marked and non-marked subgroups. The results showed over half of the regions that are both RBFOX2 bound and m^6^A marked are located at gene promoters (Fig. 1e). Similarly, m^6^A peaks that co-localize with RBFOX2-bound loci exhibit higher enrichment at gene promoters over other regions (Fig. 1e). Moreover, only 6% of genes harbouring m^6^A that are targeted by RBFOX2 are alternatively spliced upon *RBFOX2* KD in K562 cells (Extended Data Fig. 2k). These results indicate that RBFOX2 binding of m^6^A at gene promoter regions has only modest effects on splicing.

To identify the m^6^A-containing RNAs that could be recognized by RBFOX2, we used purified full-length RBFOX2 protein to pull down ribosomal-RNA (rRNA)-depleted caRNAs from K562 cells, and performed m^6^A-selective allyl chemical labelling and sequencing (m^6^A-SAC-seq)^36^, a method for transcriptome-wide quantitative mapping of m^6^A at single nucleotide resolution. RNAs pulled down by RBFOX2 harboured a higher m^6^A methylation than input (Extended Data Fig. 2l), consistent with the role of RBFOX2 as an m^6^A-binding protein. Consistently, motif search on RBFOX2-bound and m^6^A-methylated loci returned both canonical (DRACH) and non-canonical m^6^A motifs as substrates of RBFOX2 on caRNAs, with AGAUG showing the highest percentage (Fig. 1f and Extended Data Fig. 2m–p). This suggests that m^6^A recognition by RBFOX2 could be sequence context dependent. We speculated that RBFOX2 might recognize AGAUG through its RNA-recognition motif (RRM), which has UGCAUG as its canonical motif in splicing regulation^37^. To verify this hypothesis, we synthesized a pair of oligos with the non-canonical AGAUG motif (with or without m^6^A for the middle A) and performed oligo pulldown assays with our purified glutathione-S-transferase (GST)-tagged RRM domain of RBFOX2 and His-tagged maltose binding protein (MBP)–RBFOX2 (full length). As expected, both the RRM domain and full length of RBFOX2 showed higher binding affinity to m^6^A-modified oligos compared with the unmethylated ones (Fig. 1g and Extended Data Fig. 2q). Electrophoretic mobility shift assay (EMSA) further confirmed that RBFOX2 displays a higher binding affinity to methylated probes over the unmethylated ones (Extended Data Fig. 2r). Together, these results support that RBFOX2 recognizes m^6^A at gene promoters through its RRM domain.

### RBFOX2 depletion opens chromatin via m^6^A hypomethylation

**Fig. 2:**
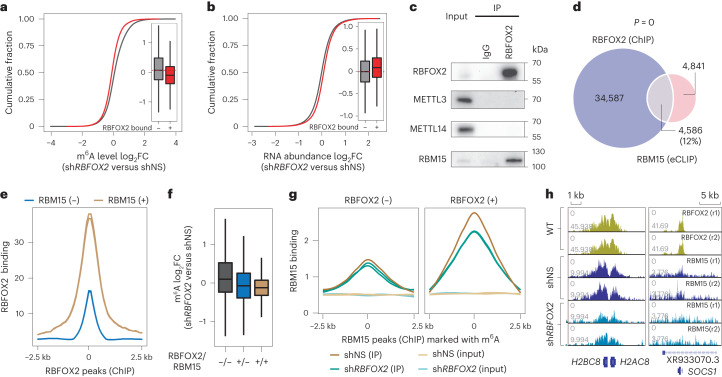
RBFOX2 recruits m^6^A MTC to gene promoter for RNA methylation. **a**, Cumulative curve and box plot (inset) of m^6^A log_2_(fold change (FC)) comparing *RBFOX2* KD (sh*RBFOX2*) versus control (shNS) K562 cells. m^6^A peaks (*N*) were categorized into two groups according to whether they overlapped with RBFOX2 peaks (+, *N* = 5,089) or not (−, *N* = 23,381) (refs. ^29,30^). **b**, Cumulative curve and box plot (inset) of RNA log_2_FC comparing *RBFOX2* KD versus control K562 cells. caRNAs (*N*) were categorized into two groups according to whether they are derived from regions with RBFOX2 binding (+, *N* = 5,089) or not (−, *N* = 23,381) (refs. ^29,30^). **c**, Western blots of the immunoprecipitated RBFOX2 from K562 cells and its interactions with METTL3, METTL14 and RBM15 after RNase A/T1 treatment. **d**, Venn diagram of overlap between RBFOX2 ChIP–seq and RBM15 eCLIP peaks in K562 cells^29,30^. Two-sided *P* value was calculated by Fisher’s exact test. **e**, Average profile of RBFOX2 binding intensity at RBFOX2 peak centres and the flanking 2.5 kb regions in K562 cells. RBFOX2 peaks were categorized into two groups according to whether they overlap with RBM15 eCLIP peaks (+) or not (−)^29,30^. **f**, Box plot of m^6^A log_2_FC comparing *RBFOX2* KD versus control K562 cells. m^6^A peaks (*N*) were categorized into three groups according to whether they overlapped with RBFOX2 or RBM15 peaks^29,30^. From left to right, *N* = 22,061, 4,049 and 1,040. **g**, Average profile of RBM15 binding intensity at RBFOX2 peak centres and the flanking 2.5 kb regions in K562 cells. RBM15 peaks were categorized into two groups according to whether they overlap with RBFOX2 (+) or not (−) (refs. ^29,30^). **h**, The integrative genomics viewer plots showing RBFOX2 ChIP–seq signals in wild-type K562 cells^29,30^, and RBM15 ChIP–seq signals in control and *RBFOX2* KD K562 cells around *H2AC8*/*H2BC8* (left) and *SOCS1* (right) gene loci. Source data

**Extended Data Fig. 3:**
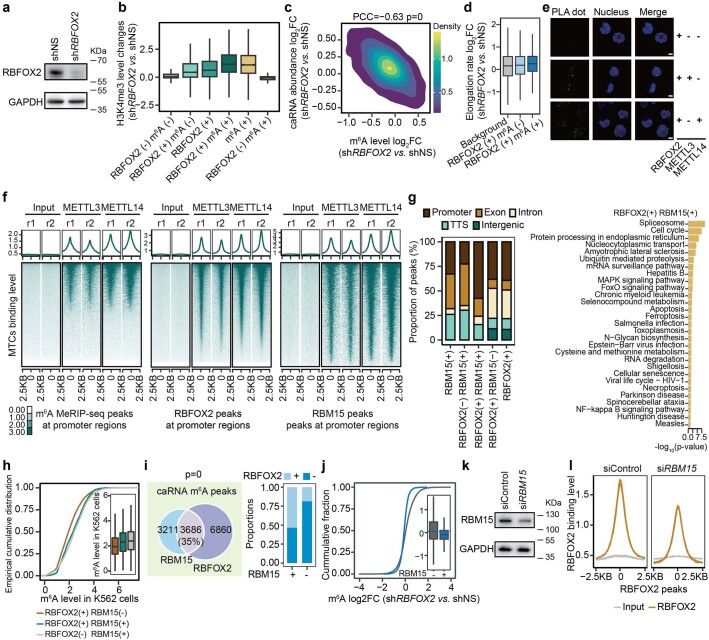
RBFOX2 recruits m^6^A MTC to gene promoter for RNA methylation installation. **a**, RBFOX2 expression in K562 cells. **b**, H3K4me3 level changes at H3K4me3 peaks (*N*) by *RBFOX2* KD in K562 cells. H3K4me3 peak regions were categorized into six groups based their overlap with RBFOX2 or m^6^A. From left to right, *N* = 9767, 11201, 14907, 3706, 3884,178. **c**, Correlation of log_2_FC between m^6^A level and caRNA abundance by *RBFOX2* KD in K562 cells. *P* value by PCC. **d**, The elongation rate changes comparing *RBFOX2* KD with control K562 cells. Transcripts (*N*) were categorized into three groups based on their overlap with RBFOX2 or m^6^A. From left to right, *N* = 130274, 13232, and 5678. **e**, *In situ* PLA detecting the close proximity (green) between RBFOX2 and METTL3 or METTL14 in K562 cells. Scale bar, 5μM. **f**, The binding intensity of METTL3 and METTL14 at m^6^A, RBFOX2 and RBM15 marked promoter regions, respectively. **g**, Distribution of RBM15 (eCLIP) or RBFOX2 (ChIP) peaks at distinct genomic regions annotated by HOMER (K562, *left* panel). Functional enrichment analysis of genes bound by both RBFOX2 and RBM15 (K562, *right* panel). One-sided *P* value by Fisher’s Exact test. **h**, m^6^A methylation level on peaks (*N*) occupied by either RBFOX2 or RBM15 (K562). From left to right, *N* = 10064, 1322 and 3187. **i**, Venn diagram of peak overlap between RBFOX2 (ChIP-seq) and RBM15 (eCLIP) in K562 cells (*left* panel). Here RBFOX2 and RMB15 peaks were required to overlap with caRNA m^6^A peaks. Barplot showing the proportion of m^6^A peaks (RBM15 bound *versus* unbound) that overlap with RBFOX2 peaks (*right* panel). Two-sided *P* value was calculated by Fisher’s exact test. **j**, m^6^A log_2_FC at m^6^A peak regions (*N*, RBM15 bound *versus* unbound) in *RBFOX2* KD *versus* control K562 cells. From left to right, *N* = 26110 and 2360. **k**, RBM15 expression in K562 cells. **l**, Average profile of RBFOX2 binding intensity at RBFOX2 peaks (K562). The depicted genome-wide data represent an integration of all samples, including two biologically independent replicates. Source data

To investigate the regulatory function of RBFOX2 in relation to m^6^A, we immunoprecipitated rRNA-depleted, m^6^A-containing caRNAs and performed methylated RNA immunoprecipitation sequencing (MeRIP-seq). We found that the methylation level of caRNAs at RBFOX2-bound loci showed a greater decrease upon *RBFOX2* KD compared with that of RBFOX2-unbound loci (Fig. 2a and Extended Data Fig. 3a). Correspondingly, RNA abundance and H3K4 trimethylation (H3K4me3) increased upon *RBFOX2* KD (Fig. 2b and Extended Data Fig. 3b). Moreover, the decrease in m^6^A methylation of caRNAs at RBFOX2-occupied loci is negatively correlated with the change in RNA abundance in *RBFOX2* KD samples (Extended Data Fig. 3c), suggesting that RBFOX2 regulates the abundance of these caRNAs through m^6^A. Next, we interrogated downstream gene transcription changes induced by *RBFOX2* KD and found that genes harbouring both caRNA m^6^A and RBFOX2-binding sites exhibited a greater increase in transcription compared with the group with RBFOX2-binding sites that do not overlap with a caRNA m^6^A site (Extended Data Fig. 3d). Taken together, we demonstrate that *RBFOX2* KD reduces caRNA m^6^A methylation, which stabilizes these caRNAs and activates downstream gene transcription.

### RBFOX2 binds RBM15 to facilitate caRNA m^6^A methylation

We next asked how the MTC components are recruited to the RBFOX2-bound genomic regions for caRNA m^6^A methylation. Although there is no physical interaction between RBFOX2 and the core heterodimer of the MTC (METTL3/METTL14), they are in close proximity inside cells (Extended Data Fig. 3e,f). In contrast, our co-immunoprecipitation (co-IP) experiments showed that RBM15, a component of MTC, physically interacts with RBFOX2, and this interaction is RNA independent (Fig. 2c). Analysis of publicly available RBM15 eCLIP-seq data indicates that about 12% of RBFOX2 genomic binding sites co-localize with RBM15 (Fig. 2d). Moreover, these overlapping regions are enriched in gene promoters (Extended Data Fig. 3g). Both RBFOX2 binding and m^6^A modification display a higher level of intensity in regions that are co-occupied by RBFOX2 and RBM15 (Fig. 2e and Extended Data Fig. 3h). Additionally, the overall co-localization between RBFOX2 and RBM15 increases three-fold (from >10% to >30%) if accounting only for the m^6^A-marked regions (Fig. 2d and Extended Data Fig. 3i). Moreover, *RBFOX2* KD caused a greater m^6^A hypomethylation at RBM15-bound sites, especially those co-occupied with RBFOX2 (Fig. 2f and Extended Data Fig. 3j), suggesting that RBFOX2 depletion could impair the recruitment of RBM15 for m^6^A installation, leading to m^6^A hypomethylation. As expected, RBM15 binding was greatly reduced at regions occupied with RBFOX2 upon *RBFOX2* KD in K562 cells (Fig. 2g,h). As RBM15 is a component of MTC, KD of *RBM15* would cause RNA m^6^A hypomethylation, which in turn reduced RBFOX2 binding at promoter sites (Extended Data Fig. 3k,l). Together, our results indicate that, by interacting with RBM15, RBFOX2 recruits the MTC for m^6^A deposition on caRNAs—particularly paRNAs. In addition, RBFOX2 preferentially binds to m^6^A-modified paRNAs, further promoting paRNA methylation.

### YTHDC1 recruits PRC2 for RBFOX2-mediated chromatin regulation

**Fig. 3:**
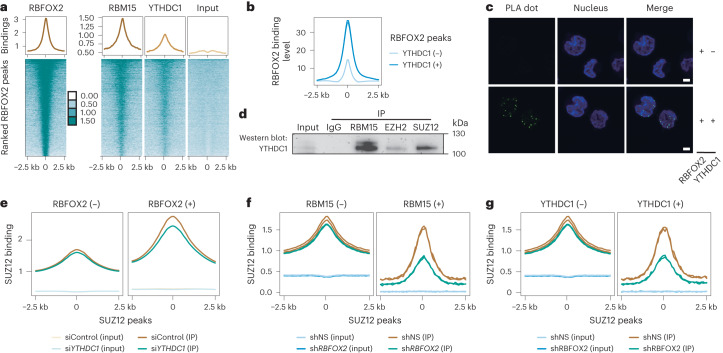
RBFOX2 regulates chromatin state through the m^6^A/RBM15/YTHDC1/PRC2 axis. **a**, Average profile (top) and heat map (bottom) showing RBFOX2, RBM15, YTHDC1 and the corresponding input signal at RBFOX2 peak centres and the flanking 2.5 kb regions in K562 cells. **b**, Average profile of RBFOX2 binding intensity at RBFOX2 peak centres and the flanking 2.5 kb regions in K562 cells. RBFOX2 peaks were categorized into two groups according to whether they overlap with YTHDC1 (+) or not (−). **c**, In situ PLA^38^ assay detecting the interaction (green) between RBFOX2 and YTHDC1 in K562 cells. Nuclei were stained with Hoechst 33342 (blue). Scale bar, 5 µm. **d**, Western blots of the immunoprecipitated RBM15, EZH2 and SUZ12, respectively, from K562 cells and their interactions with YTHDC1 after RNase A/T1 treatment. **e**, Average profile showing SUZ12 binding signal at their peak centres and the flanking 2.5 kb regions in *YTHDC1* KD (si*YTHDC1*) versus control (siControl) K562 cells. SUZ12 peaks were categorized into two groups according to whether they overlap with RBFOX2 (+) or not (−). **f**,**g**, Average profile showing SUZ12 binding signal at their peak centres and the flanking 2.5 kb regions in *RBFOX2* KD versus control K562 cells. SUZ12 peaks were categorized into two groups according to whether (+) or not (−) they were overlapped with RBM15 (**f**) or YTHDC1 (**g**). The depicted genome-wide data represent an integration of all samples, including two biologically independent replicates. Source data

**Extended Data Fig. 4:**
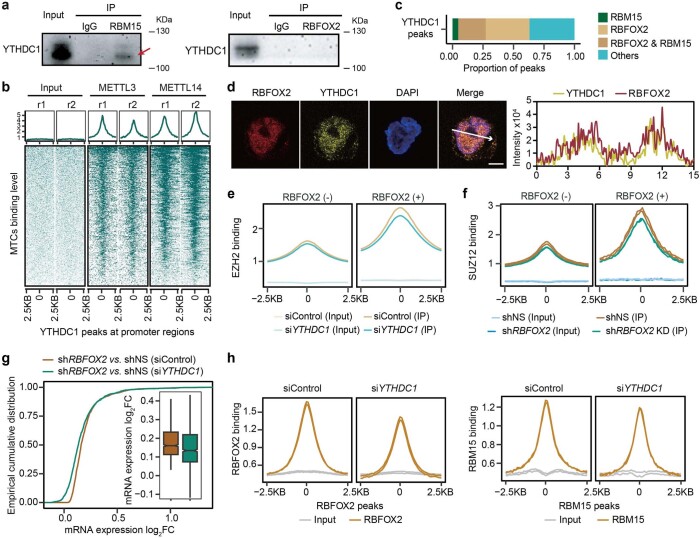
RBFOX2 regulates chromatin state through the m^6^A/RBM15/YTHDC1/PRC2 axis. **a**, Western blots of immunoprecipitated RBFOX2 and RBM15 from K562 cells and their interactions with YTHDC1 after RNase A/T1 treatment. **b**, Heatmap showing the binding intensity of METTL3 and METTL14 at YTHDC1-marked promoter regions. **c**, Barplot showing the proportion of YTHDC1 peaks overlapping with RBFOX2 or RMB15 in K562 cells. **d**, Immunofluorescence images (*left* panel) and intensity profile (*right* panel) of RBFOX2 protein (red) and YTHDC1 protein (yellow) showing their colocalization in K562 cells. Scale bar, 5μM. **e**, Average profile showing EZH2 level at their peak centers and the flanking 2.5 kb regions in *YTHDC1* KD (si*YTHDC1*) *versus* control (siControl) K562 cells. EZH2 peaks were categorized into RBFOX2 bound (+) and unbound (−) subgroups. **f**, Average profile showing SUZ12 binding signals at their peak centers and the flanking 2.5 kb regions in *RBFOX2* KD *versus* control K562 cells. SUZ12 peaks were categorized into RBFOX2 bound (+) and unbound (−) subgroups. **g**, Cumulative distribution of gene expression changes in siControl K562 cells upon *RBFOX2* knockdown *versus* control, as well as in *YTHDC1* knockdown K562 cells upon *RBFOX2* knockdown *versus* control. The displayed genes *(N* = 1221) were those that were upregulated upon *RBFOX2* knockdown in siControl K562 cells and bound by RBFOX2 at their promoter regions. **h**, Average profile of RBFOX2 (*left* panel) or RBM15 (*right* panel) at their respective peak centers and the flanking 2.5 kb regions in Control (siControl) *versus YTHDC1* knockdown (si*YTHDC1*) K562 cells. Source data

We next investigated the mechanism underlying chromatin repression affected by RBFOX2 in relation to m^6^A. YTHDC1, an m^6^A reader protein in the nucleus, interacts with various histone modifiers related to chromatin silencing^1,25–27^ in an m^6^A-dependent manner. Our co-IP experiments showed that YTHDC1 directly interacts with RBM15 but not with RBFOX2 (Extended Data Fig. 4a,b). However, more than half of YTHDC1 genomic binding sites co-localize with RBFOX2 (Fig. 3a and Extended Data Fig. 4c), and RBFOX2 displays higher binding intensity at the YTHDC1 occupied regions (Fig. 3b). Using in situ proximity ligation assay (PLA)^38^ and protein immunofluorescence (IF), we demonstrated that RBFOX2 is located in close proximity to YTHDC1 (Fig. 3c and Extended Data Fig. 4d), even though the two proteins do not physically interact with each other. These results suggest a model of YTHDC1 recruitment by RBM15 to the RBFOX2-occupied loci.

YTHDC1 is reported to physically interact with SETDB1 for heterochromatin silencing in mouse embryonic stem cells^25,26,28^. However, its role in other cell types, particularly cancer cells, has yet to be investigated. RBFOX2 has been reported to recruit the polycomb repressive complex 2 (PRC2, which is composed of EED, SUZ12 and EZH1/2) for trimethylation of histone H3 at lysine 27 (H3K27me3) and causes transcriptional repression^34^, although the detailed pathway was unclear. We wondered whether the RBFOX2/RBM15 interaction could lead to chromatin regulation through potential recruitment of PRC2. Indeed, we found a direct interaction between YTHDC1 and the PRC2 complex (Fig. 3d). To further investigate the recruitment of PRC2 by YTHDC1, we performed SUZ12 and EZH2 ChIP–seq in *YTHDC1* KD K562 cells. PRC2 binding showed a notable decrease at the regions bound by RBFOX2, whereas no obvious changes were observed at unbound regions (Fig. 3e and Extended Data Fig. 4e), suggesting that YTHDC1 facilitates PRC2 recruitment to the RBFOX2-bound regions. Furthermore, SUZ12 level showed minor global changes upon *RBFOX2* KD in K562 cells. However, at regions co-occupied by either RBM15 or YTHDC1, SUZ12 binding was dramatically reduced upon *RBFOX2* KD (Fig. 3f,g and Extended Data Fig. 4f). Moreover, *YTHDC1* KD partially rescued the upregulation of gene expression induced by *RBFOX2* KD (Extended Data Fig. 4g). It is noteworthy that the KD of *YTHDC1* resulted in a reduction of the genomic binding of both RBFOX2 and RBM15, suggesting that YTHDC1 may be necessary for the proper recruitment or retention of RBFOX2/RBM15 at gene promoter regions (Extended Data Fig. 4h). Altogether, we show that RBFOX2 regulates chromatin state through the RBM15/YTHDC1/PRC2 axis.

### RBFOX2 depletion promotes leukaemia cell differentiation

**Fig. 4:**
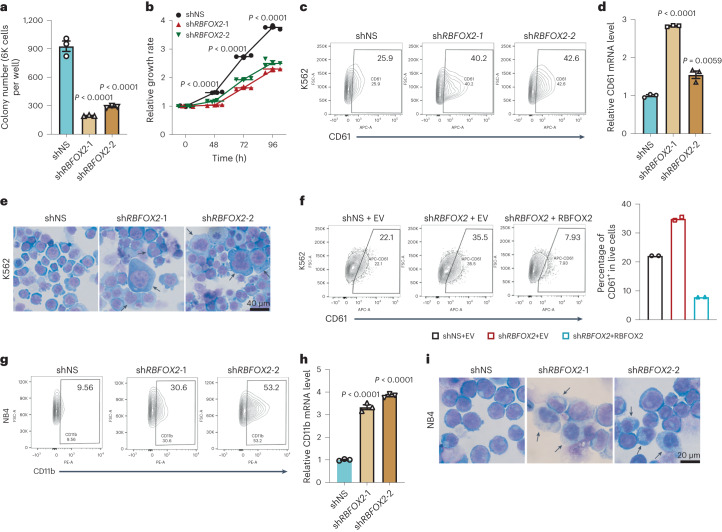
RBFOX2 depletion promotes the differentiation of leukaemia cells. **a**, Colony-forming assays of K562 cells transduced with control (shNS) or two *RBFOX2* shRNAs (sh*RBFOX2*-1 and sh*RBFOX2*-2). *n* = 3. **b**, Effects of *RBFOX2* KD on K562 cell growth. *n* = 3. *P* value was calculated by two-way ANOVA, Dunnett’s multiple comparisons test. **c**, Flow cytometric analysis of CD61^+^ cell populations in K562 cells transduced with shNS or *RBFOX2* shRNAs (sh*RBFOX2*-1 and sh*RBFOX2*-2). **d**, Relative expression of CD61 in control (shNS) and *RBFOX2* KD (sh*RBFOX2*-1 and sh*RBFOX2*-2) K562 cells. *n* = 3. Two-sided *P* value was calculated by Student’s *t*-test. **e**, Wright-Giemsa staining of cytospin slides of K562 cells transduced with shNS or *RBFOX2* shRNAs (sh*RBFOX2*-1 and sh*RBFOX2*-2). Arrows indicate differentiated cells. Scale bar, 40 µm. **f**, Flow cytometric analysis of CD61^+^ cell populations in control group (shNS + EV, control shRNA (shNS) with empty vector (EV)), *RBFOX2* KD group (sh*RBFOX2* + EV, *RBFOX2* KD with EV) and RBFOX2 rescue group (sh*RBFOX2* + RBFOX2, *RBFOX2* KD with RBFOX2 overexpression) in K562 cells (left), and statistics from three biological replicates (right). EV or RBFOX2 overexpressed cells were labelled with green fluorescent protein (GFP), shNS or sh*RBFOX2* transduced cells expressed mCherry. GFP^+^mCherry^+^ cells represent positively double transduced cells. *n* = 2. **g**, Flow cytometric analysis of CD11b^+^ cell populations in control (shNS) and *RBFOX2* KD (sh*RBFOX2*-1 and sh*RBFOX2*-2) NB4 cells. **h**, Relative expression of CD11b in control (shNS) and two *RBFOX2* KD (sh*RBFOX2*-1 and sh*RBFOX2*-2) NB4 cells. *n* = 3. **i**, Wright-Giemsa staining of cytospin slides of NB4 cells transduced with shNS or *RBFOX2* shRNAs (sh*RBFOX2*-1 and sh*RBFOX2*-2). Arrows indicate differentiated cells. Scale bar, 20 µm. *n*, biologically independent samples. Data are presented as mean ± standard error of the mean. For **a** and **h**, *P* values were calculated by one-way ANOVA, Dunnett’s multiple comparisons test. Source data

**Extended Data Fig. 5:**
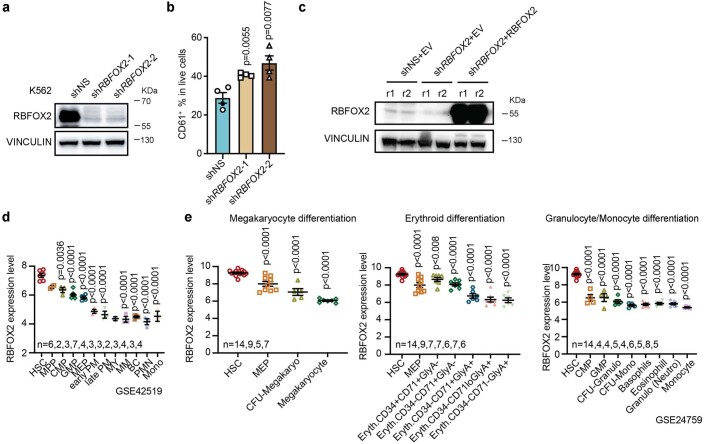
*RBFOX2* knockdown inhibits K562 cell growth and promotes K562 cell differentiation towards megakaryocyte lineage. **a**, Western blot of RBFOX2 in K562 cells transfected with shNS or *RBFOX2* shRNAs (sh*RBFOX2*-1 and sh*RBFOX2*-2). **b**, Percentages of cells with CD61^+^ staining in K562 cells transduced with shNS or *RBFOX2* shRNAs (sh*RBFOX2*-1 and sh*RBFOX2*-2). *n* = 10000 cells examined over 4 biologically independent experiments. **c**, Western blot showing RBFOX2 expression in control group (shNS+EV, control shRNA (shNS) with empty vector (EV)), *RBFOX2* KD group (sh*RBFOX2* + EV, *RBFOX2* KD with EV) and RBFOX2 rescue group (sh*RBFOX2* + RBFOX2, *RBFOX2* KD with RBFOX2 overexpression) in K562 cells. **d-e**, Relative expression of *RBFOX2* during hematopoietic cell differentiation into different lineages based on GSE42519 (***d***) and GSE24759 (***e***) datasets. The sample size shown in (***d***) and (***e***) are biologically independent human samples. Data are presented as mean values +/− SEM. Two-sided *P* values by Student’s t-test. Source data

While investigating the role of RBFOX2 in K562 (a chronic myelogenous leukaemia cell line) cells, we found that RBFOX2 depletion led to a noticeably reduced colony forming ability and impaired cell growth (Fig. 4a,b and Extended Data Fig. 5a). Interestingly, *RBFOX2* KD greatly promoted the differentiation of K562 cells towards the megakaryocyte lineage, as shown by the expression of megakaryocyte marker CD61^+^ and cell morphology changes (Fig. 4c–e and Extended Data Fig. 5b). Furthermore, these effects were reversed when RBFOX2 was reintroduced into *RBFOX2*-depleted cells (Fig. 4f and Extended Data Fig. 5c). Haematopoietic cells commit to producing mature blood cells through the megakaryocyte–erythroid progenitor lineage and granulocyte/monocyte progenitor (myeloid lineage)^39^. We wondered whether RBFOX2 is also involved in regulating myeloid lineage differentiation events in addition to megakaryocyte/erythroid lineage differentiation. We examined expression of *RBFOX2* in different haematopoietic cell lineages. Of note, *RBFOX2* is expressed at a high level in haematopoietic stem cells (HSCs) but is markedly downregulated during both megakaryocyte/erythroid and granulocyte/monocyte differentiation (Extended Data Fig. 5d,e), suggesting RBFOX2 might be involved in myeloid lineage commitment.

**Extended Data Fig. 6:**
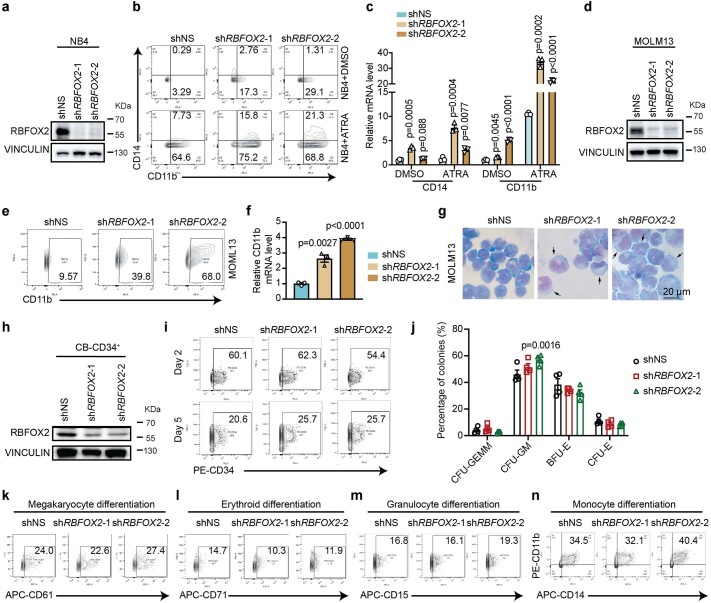
*RBFOX2* knockdown promotes the differentiation of leukemia cells. **a**, Western blot assay to quantify RBFOX2 knockdown efficiency in NB4 cells. **b-c**, Flow cytometric analysis of CD11b^+^ cell populations (***b***), and relative gene expression of CD14 and CD11b with qPCR quantification (***c***) in control (shNS) and *RBFOX2* KD (sh*RBFOX2*-1 and sh*RBFOX2*-2) NB4 cells after treatment with 500 nM ATRA for 72 hr as compared to DMSO-treated cells. *n* = 3. **d**, Western blot assay to quantify RBFOX2 knockdown efficiency in MOLM13 cells. **e-f**, Flow cytometric analysis of CD11b^+^ cell populations (***e***) and CD11b mRNA expression level (***f***) in control (shNS) and *RBFOX2* KD (sh*RBFOX2*-1 and sh*RBFOX2*-2) MOLM13 cells. *n* = 3. **g**, Wright-Giemsa staining of cytospin slides of control (shNS) and *RBFOX2* KD (sh*RBFOX2*-1 and sh*RBFOX2*-2) MOLM13 cells. Arrows indicate differentiated cells. Scale bar, 20 µm. **h**, Western blots showing the knockdown efficiency of RBFOX2 in human cord blood-derived CD34^+^ (CB-CD34^+^) cells. **i**, Flow cytometric analysis of the percentage of CD34^+^ cell population in control (shNS) and *RBFOX2* KD (sh*RBFOX2*-1 and sh*RBFOX2*-2) CB-CD34^+^ cells. Day 2 and Day 5 represent the number of days post-transduction. **j**, Quantification of the percentage of different hematopoietic lineage progenitor colonies in control (shNS) and *RBFOX2* KD (sh*RBFOX2*-1 and sh*RBFOX2*-2) CB-CD34^+^ cells. *n* = 4. *P* values were calculated by two-way ANOVA, Dunnett’s multiple comparisons test. **k-n**, Flow cytometric analysis of megakaryocyte (**k**), erythroid (**l**), granulocyte (**m**) and monocyte (**n**) differentiation of CB-CD34^+^ cells (five days post-transduction) transfected with shNS or *RBFOX2* shRNAs (sh*RBFOX2*-1 and sh*RBFOX2*-2). *n*, biologically independent samples. Data are presented as mean values +/− SEM. For **c** and **f**, two-sided *P* values by Student’s t-test. Source data

As AML cell lines are usually blocked at different stages during myeloid differentiation, they are widely used as models to study myeloid differentiation^40^. We knocked down RBFOX2 in human acute promyelocytic leukaemia cell line NB4 to evaluate its role in myeloid differentiation (Extended Data Fig. 6a). Indeed, RBFOX2 depletion significantly promoted myeloid differentiation and resulted in a marked increase in expression of CD11b, a myeloid differentiation marker (Fig. 4g,h). Cell morphology studies further confirmed the elevated myeloid differentiation in the *RBFOX2*-depleted NB4 cells (Fig. 4i). Additionally, the depletion of *RBFOX2* further sensitizes NB4 cells to all-*trans* retinoic acid (ATRA)-induced granulocyte differentiation (Extended Data Fig. 6b,c). Similar results were obtained using the human acute monocytic leukaemia cell line MOLM13 (Extended Data Fig. 6d–g). We did not observe any enhancement in colony formation and differentiation upon depletion of *RBFOX2* in CD34^+^ cells derived from normal human umbilical cord blood (UCB-CD34^+^ cells) (Extended Data Fig. 6h–n). Altogether, these results suggest that depletion of RBFOX2 promotes myeloid differentiation exclusively in leukaemia.

### RBFOX2 depletion impairs leukaemia progression in vivo

**Fig. 5:**
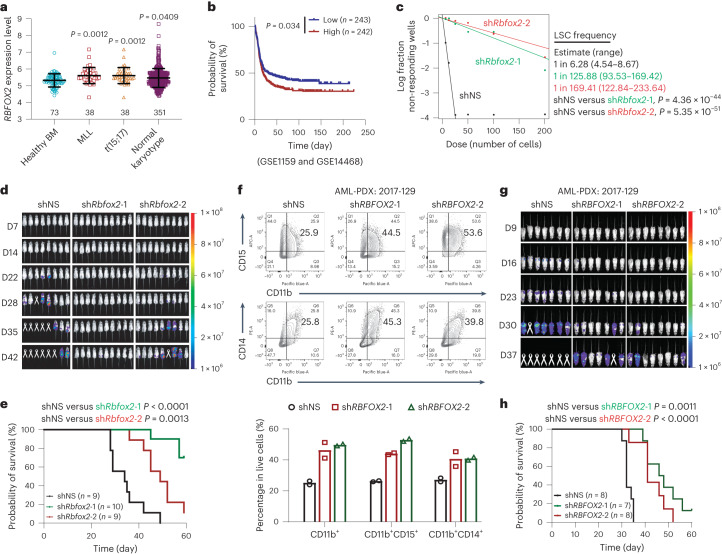
RBFOX2 is aberrantly expressed in AML and RBFOX2 depletion impairs leukaemia progression in vivo. **a**, Expression levels of *RBFOX2* in patients with primary AML bearing chromosomal translocations and those in bone marrow (BM) HSCs collected from healthy donors (healthy BM) (GSE13159 (ref. ^49^)). *n* = 73 for healthy BM, *n* = 38 for MLL, *n* = 38 for *t*(15;17) and *n* = 351 for normal karyotype. Two-sided *P* values were calculated by Student’s *t*-test. **b**, Kaplan–Meier survival analysis in GSE1159 (ref. ^50^) and GSE14468 (ref. ^51^) dataset (*n* = 485). The patients were divided into two groups of equal size based on *RBFOX2* levels. **c**, In vitro LDAs. Logarithmic plot showing the percentage of non-responding wells at different doses. Non-responding wells are wells not containing colony-forming cells. The estimated LSC/LIC frequency is calculated by ELDA and shown on the right. **d**, Bioluminescence imaging of mice transplanted with luciferase-expressing MOLM13 cells transfected with control (shNS) and *RBFOX2* shRNAs (sh*RBFOX2*-1 and sh*RBFOX2*-2), respectively. D, day. **e**, Kaplan–Meier survival curves of recipient mice transplanted with control (*n* = 9) and two *RBFOX2* KD (*n* = 10 for sh*RBFOX2-*1 and *n* = 9 for sh*RBFOX2-*2) MOLM13 cells. **f**, Flow cytometric analysis (top) and quantification (bottom) of CD11b^+^ cell populations in control and *RBFOX2* KD AML-PDX cells (2017-129 (ref. ^44^)). *n* = 2. **g**, Bioluminescence imaging of mice transplanted with luciferase-expressing AML-PDX cells (2017-129 (ref. ^44^)) transduced with shNS and *RBFOX2* shRNAs (sh*RBFOX2*-1 and sh*RBFOX2*-2), respectively. **h**, Kaplan–Meier survival curves of recipient mice transplanted with control (*n* = 8) and *RBFOX2* KD (*n* = 7 for sh*RBFOX2*-1 and *n* = 8 for sh*RBFOX2*-2) AML-PDX cells (2017-129 (ref. ^44^)). *n*, biologically independent samples. Data are presented as mean ± standard error of the mean. For **b**, **c**, **e** and **h**, *P* values were calculated by the log-rank test. Source data

**Extended Data Fig. 7:**
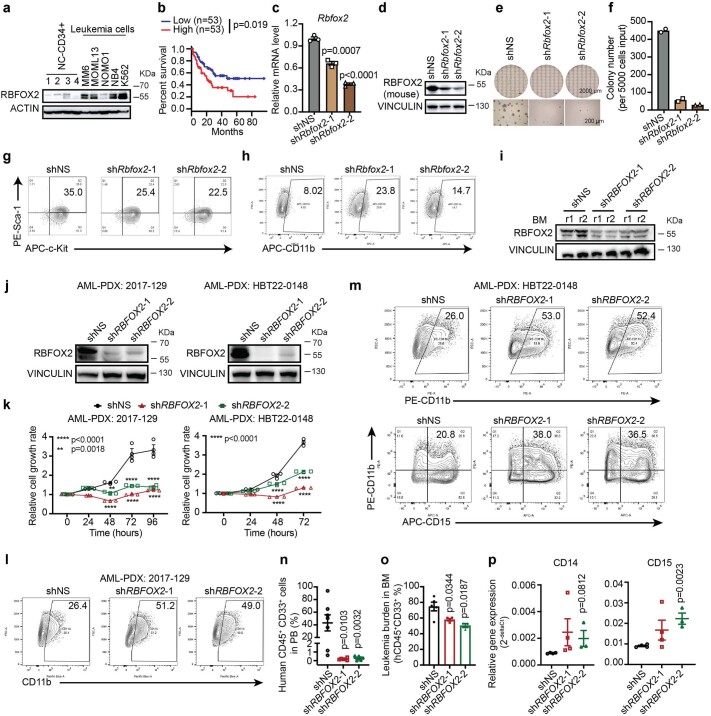
*RBFOX2* knockdown blocks LSC self-renewal, and impairs AML progression *in vivo*. **a**, Western blot of RBFOX2 protein in normal controls (human bone marrow CD43^+^) and AML cell lines. **b**, Kaplan-Meier survival analysis in TCGA-AML dataset (*n* = 106) using GEPIA. The patients were divided into two groups of equal size based on *RBFOX2* levels. *n*, biologically independent patients. *P* value was detected by the log-rank test. **c-d**, Relative *Rbfox2* gene expression quantified by qPCR (***c****, n* = 3) and western blot assay (***d***) in control (shNS) and *Rbfox2* KD (sh*Rbfox2*-1 and sh*Rbfox2-*2) mouse MLL-AF9 (MF9) cells. **e-f**, Image (***e***) and quantification (***f****, n* = 2) of effects of *Rbfox2* knockdown on the colony-forming of mouse MA9 AML cells. **g**, Flow cytometric analysis of LSC populations in mouse MA9 leukemia cells. **h**, Flow cytometric analysis of CD11b^+^ cell populations in mouse MA9 leukemia cells. **i**, Western blot of RBFOX2 protein in control (shNS) and *RBFOX2* KD (sh*RBFOX2*-1 and sh*RBFOX2-2*) in *in vivo* bone marrow samples. **j**, Western blot showing RBFOX2 expression in control and *RBFOX2* KD (sh*RBFOX2*-1 and sh*RBFOX2*-2) AML-PDX cells. **k**, Effects of *RBFOX2* knockdown (shNS [*n* = 4], sh*RBFOX2*-1 [*n* = 4] and sh*RBFOX2*-2 [*n* = 3]) on AML-PDX cell growth. *P* value was calculated by one-way ANOVA, Dunnett’s multiple comparisons test. **l**, Flow cytometric analysis of CD11b^+^ cell populations in control and *RBFOX2* KD AML-PDX cell (2017-129). **m**, Flow cytometric analysis of CD11b^+^ (*top* panel) or CD11b^+^ and CD15^+^ (*bottom* panel) cell populations in control and *RBFOX2* KD AML-PDX cell (HBT22-0148). **n**, The hCD45 and hCD33 double positive cells (CD45^+^CD33^+^) were utilized to determine the engraftment of human AML-PDX cells (2017-129^44^) in recipient mice on day 34 post xeno-transplantation (shNS [*n* = 8], sh*RBFOX2*-1 [*n* = 7] and sh*RBFOX2*-2 [*n* = 9]). **o**, Quantification of leukemia burden in control (shNS [*n* = 5]) and *RBFOX2* KD (sh*RBFOX2*-1 [*n* = 4] and sh*RBFOX2*-2 [*n* = 3]) bone marrow (BM) cells. **p**, Relative gene expression of CD14 and CD15 in control (shNS [*n* = 4)) and *RBFOX2* KD (shRBFOX2-1 [n = 4] and shRBFOX2-2 [*n* = 3]) BM cells. n, biologically independent mice. Data are presented as mean values +/− SEM. For **c, n**, **o** and **p**, two-sided *P* values by Student’s t-test. Source data

AML is a fatal form of haematopoietic malignancy, characterized by the clonal expansion and differentiation blockage of myeloid progenitor cells^41,42^. The blockage in myeloid differentiation by RBFOX2 as we have demonstrated suggests its oncogenic role in AML. We profiled *RBFOX2* mRNA expression levels in a large cohort of patients with AML (GSE13159, *n* = 500) and observed significantly higher *RBFOX2* expression in multiple subtypes of AML compared with healthy bone marrow controls, which was validated by our western blot assay (Fig. 5a and Extended Data Fig. 7a). Moreover, higher *RBFOX2* expression is associated with shorter overall survival in patients with AML (Fig. 5b and Extended Data Fig. 7b).

We further examined the role of RBFOX2 in the self-renewal of leukaemic stem cells (LSCs)/leukaemic initiating cells (LICs), as drug resistance and relapse have been linked with the existence of LSCs/LICs in patients with AML^41,43^. In MLL-AF9 (MA9) leukaemia mice, KD of *Rbfox2* greatly inhibited colony-forming capacity of leukaemic bone marrow blast cells, reduced LSC population and promoted their differentiation (Extended Data Fig. 7c–h), suggesting RBFOX2 is required for LSC/LIC maintenance. To quantitatively assess the effect of *Rbfox2* depletion on LSC/LIC self-renewal, we conducted in vitro limiting dilution assays (LDAs) and found that *Rbfox2* KD significantly decreased LSC/LIC frequency in mouse MA9 cells (1/125.88 and 1/169.41 in *Rbfox2* KD versus 1/6.28 in control cells, *P* < 0.001) (Fig. 5c). Strikingly, KD of *RBFOX2* markedly impaired human AML progression in immunocompromised recipient mice and significantly prolonged mice survival (Fig. 5d,e and Extended Data Fig. 7i). We further employed a patient-derived xenograft (PDX) AML mouse model (with a relapsed AML patient sample, 2017-129 and HBT22-0148) (ref. ^44^). Consistent with our findings in the xenograft model using AML cell lines, *RBFOX2* KD significantly inhibited cell growth, promoted myeloid differentiation and substantially prolonged mouse survival in the AML PDX model (Fig. 5f–h and Extended Data Fig. 7j–n). We consistently observed a reduced leukaemia burden, which might be attributed to the promotion of differentiation resulting from *RBFOX2* depletion (Extended Data Fig. 7o,p). Collectively, these results suggest that RBFOX2 is required for AML cell survival and leukaemia maintenance, which it may facilitate by promoting LSC/LIC self-renewal and inhibiting AML cell differentiation.

### RBFOX2 suppresses *TGFB1* to drive AML tumourigenesis

**Fig. 6:**
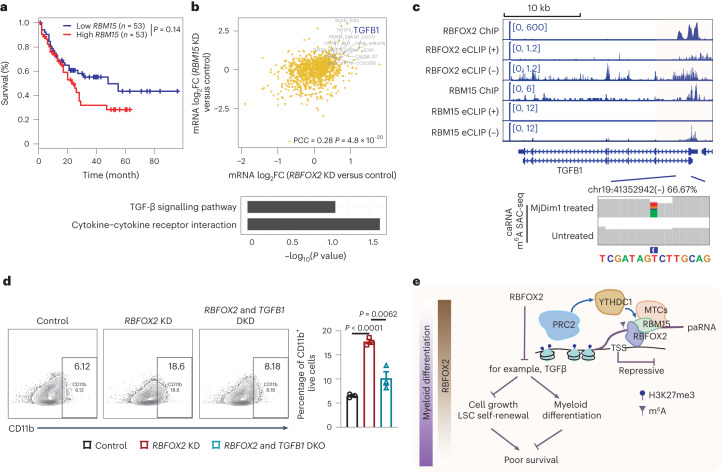
RBFOX2 suppresses *TGFB1* transcription to promote tumourigenesis in human AML. **a**, Kaplan–Meier survival analysis in TCGA-AML dataset (*n* = 106) using GEPIA^52^. The patients were divided into two groups according to gene expression level of *RBM15*. *P* value was determined by the log-rank test. **b**, Top: correlation of gene expression log_2_FC between *RBFOX2* KD versus control and *RBM15* KD versus control K562 cells^29,30^. Here genes are required to be bound by both RBFOX2 and RBM15, and genes modified with m^6^A are highlighted. Bottom: bar plot (bottom) showing the functional enrichment analysis of genes harbouring m^6^A and regulated by RBFOX2 and RBM15. PCC was employed for correlation analysis, and the corresponding *P* value was obtained. **c**, The integrative genomics viewer plots showing the binding profiles of RBFOX2 and RBM15, and m^6^A level in K562 cells around the *TGFB1* gene locus. In eCLIP-seq, ‘(+)’ represents the reads from the forward strand and ‘(−)’ represents the reads from the reverse strand to the genome. **d**, A representative flow cytometric (left) and quantification (right) analysis of CD11b^+^ cell populations in control, *RBFOX2* KD and *RBFOX2* & *TGFB1* double KD NB4 cells, respectively. *n* = 3 biologically independent samples. Data are presented as mean ± standard error of the mean. Two-sided *P* values were calculated by Student’s *t*-test. **e**, A proposed model depicts RBFOX2 regulation on tumourigenesis through the m^6^A/RBM15/YTHDC1/PRC2 axis. Source data

**Extended Data Fig. 8:**
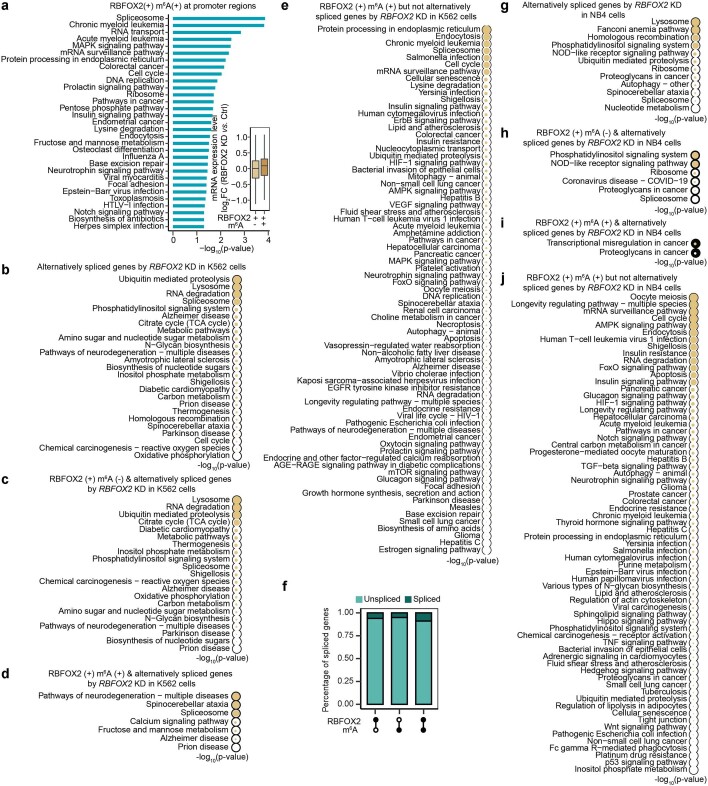
RBFOX2 regulation of m^6^A is not significantly related to its role in regulating alternative splicing. **a**, Functional enrichment analysis of genes with their promoters bound by RBFOX2 and marked with both H3K4me3 and m^6^A. Inset boxplot shows gene expression level log_2_FC comparing *RBFOX2* KD with control K562 cells. Genes with RBFOX2 binding and H3K4me3 modification were categorized into two subgroups according to whether they were marked with m^6^A (+) or not (−). *n* = 2 biologically independent samples. RBFOX2 binding are from ENCODE project, and m^6^A MeRIP-seq and H3K4me3 ChIP-seq are from this study. **b**, Enrichment analysis of alternatively spliced genes upon *RBFOX2* knockdown in K562 cells. **c-d**, Enrichment analysis of RBFOX2 bound and alternatively spliced genes upon *RBFOX2* knockdown in K562 cells. Alternatively spliced genes bound by RBFOX2 were categorized into two groups according to whether those genes were methylated by m^6^A (RBFOX2(+) m^6^A(+)) or not (RBFOX2(+) m^6^A(−)). **e**, Enrichment analysis of genes harboring both RBFOX2 binding and m^6^A methylation, but which were not alternatively spliced upon *RBFOX2* knockdown in K562 cells. **f**, Barplot showing the ratios of alternatively spliced genes by *RBFOX2* knockdown in NB4 cells. **g**, Enrichment analysis of alternatively spliced genes upon *RBFOX2* knockdown in NB4 cells. **h-i**, Enrichment analysis of RBFOX2 bound and alternatively spliced genes upon *RBFOX2* knockdown in NB4 cells. RBFOX2 bound and alternatively spliced genes were categorized into two groups according to whether those genes were methylated by m^6^A (RBFOX2(+) m^6^A(+)) or not (RBFOX2(+) m^6^A(−)). **j**, Enrichment analysis of genes harboring both RBFOX2 binding and m^6^A methylation, but which were not alternatively spliced upon *RBFOX2* knockdown in NB4 cells. For **b**-**e** and **g-j**, one-sided *P* values were calculated using Fisher’s Exact test, and the size of the circle represents the level of significance, with larger circles indicating greater significance and smaller circles indicating lower significance.

**Extended Data Fig. 9:**
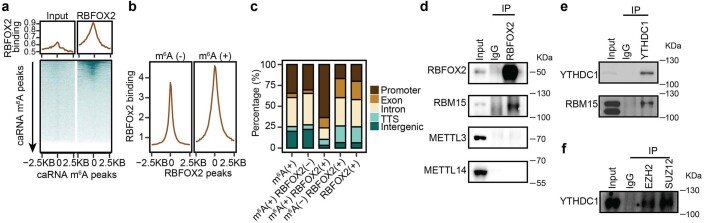
RBFOX2 functions as an m^6^A regulator in AML cells. **a**, Average profile (*top* panel) and heatmap (*bottom* panel) showing RBFOX2 binding intensity at the m^6^A peak centers and the flanking 2.5 kb regions in NB4 cells. **b**, Average profile of RBFOX2 binding intensity at the RBFOX2 peak centers and the flanking 2.5 kb regions in NB4 cells. RBFOX2 peaks were categorized into two groups according to whether they overlap with m^6^A (+) or not (−). **c**, Distribution of m^6^A or RBFOX2 peaks at distinct genomic regions including promoter, exonic, intronic, transcription termination sites (TTS) and intergenic regions annotated by HOMER in NB4 cells. (+) sign represents regions harboring m^6^A (+) or bound by RBFOX2 (+), while the sign (−) indicates the absence of m^6^A (−) or RBFOX2 (−). **d**, Western blots of the immunoprecipitated RBFOX2 from NB4 cells and its interaction with METTL3, METTL14 and RBM15 after RNase A/T1 treatment, respectively. **e**, Western blots of the immunoprecipitated YTHDC1 from NB4 cells and its interaction with RBM15 after RNase A/T1 treatment. **f**, Western blots of the immunoprecipitated EZH2 or SUZ12 from NB4 cells, and their interactions with YTHDC1 after RNase A/T1 treatment. Source data

**Extended Data Fig. 10:**
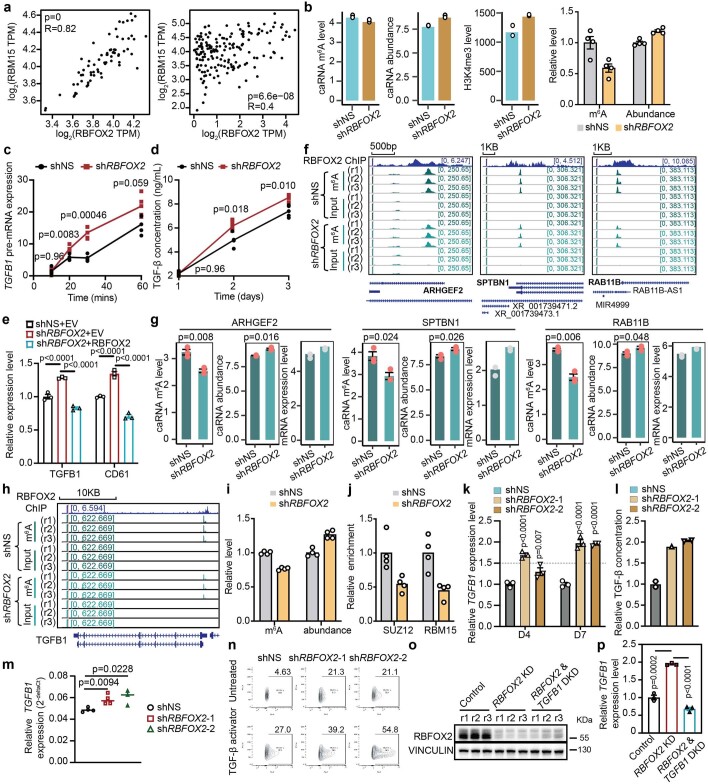
RBFOX2 regulates leukemia differentiation through TGF-β signaling pathway. **a**, Correlation of gene expression between *RBOFX2* and *RBM15* in Leukemia cell line (CML) (*left* panel) from GTEx and in AML patients (*right* panel) from TCGA using GEPIA. *P* values by PCC. **b**, m^6^A level, caRNA abundance and H3K4me3 level at the *TGFB1* promoter (K562, the first three panels with high-throughput sequencing data, *n* = 2; the forth panel with RT-qPCR, *n* = 4 technical replicates). **c-d**, Nascent RNA synthesis (**c**) and ELISA (**d**) of *TGFB1* in K562 cells (*n* = 4, two technical replicates over two biological replicates). **e**, Gene expression level of *TGFB1* and CD61 in shNS+EV, sh*RBFOX2* + EV and sh*RBFOX2* + RBFOX2 K562 cells. *n* = 3. **f-g**, Decreased m^6^A levels (**f** and **g**), increased RNA abundances at paRNA loci (*n* = 3, **g**) and increased mRNA expression (*n* = 2, **g**) of *ARHGEF2*, *SPTBN1*and *RAB11B* upon *RBFOX2 KD* in NB4 cells, respectively. **h**, The RBFOX2 binding and m^6^A level around the *TGFB1* gene locus in NB4 cells. **i**, The m^6^A level and caRNA abundance at the *TGFB1* promoter in NB4 cells (RT-qPCR, *n* = 4 technical replicates). **j**, SUZ12 or RBM15 binding at the *TGFB1* promoter in NB4 cells (ChIP qPCR, *n* = 4 technical replicates). **k**, Gene expression level of *TGFB1* in NB4 cells. *n* = 3. **l**, TGF-β ELISA assay in NB4 cells (shNS [*n* = 2], sh*RBFOX2*-1 [*n* = 1] and sh*RBFOX2*-2 [*n* = 2]). **m**, Relative gene expression of human *TGFB1* in the bone marrow of leukemia mice from AML-PDX cells (2017-129) (shNS [*n* = 4], sh*RBFOX2*-1 [*n* = 4] and sh*RBFOX2*-2 [*n* = 3]). **n**, Flow cytometric analysis of CD11b^+^ cells in NB4 cells treated with TGF-*β* activator (0.6 ng/mL) or not (Untreated). **o-p**, The expression level of RBFOX2 (protein, **o**) and *TGFB1* (mRNA, **p**) in control, *RBFOX2* KD, and *RBFOX2 & TGFB1* double KD (DKD) NB4 cells. *n* represents biologically independent samples, unless otherwise stated. For **b**, **e**, **g**, **i**, **j**, **k**, **m** and **p**, mean values +/− SEM; for **c** and **d**, mean values +/− SD. For **g**, **m** and **p**, two-sided *P* values by Student’s t-test; for **e** and **k**, *P* values by two-way ANOVA, Dunnett’s multiple comparisons test. Source data

Functional analysis of genes harbouring m^6^A and bound by RBFOX2 at their promoters highlights pathways related with both chronic and acute myeloid leukaemia (Extended Data Fig. 8a). Notably, these pathways were not observed for genes that are alternatively spliced upon *RBFOX2* depletion (Extended Data Fig. 8b–j). As we showed in K562 cells, the m^6^A/RBM15/YTHDC1/PRC2 axis regulates transcription of genes important to myeloid differentiation in AML cells (Extended Data Fig. 9). It is worth noting that the expression levels of *RBM15* and *RBFOX2* are positively correlated in patients with AML, and higher *RBM15* expression is associated with shorter overall survival, similar to that of *RBFOX2* (Fig. 6a and Extended Data Fig. 10a), indicating an interplay between RBFOX2 and m^6^A deposition/RBM15 binding on effects in AML.

To better characterize how RBFOX2 regulates myeloid leukaemia via m^6^A, we correlated the expression changes of the genes co-occupied by RBFOX2 and RBM15 in either *RBFOX2* KD or *RBM15* KD K562 cells, and found a positive correlation, as expected (Fig. 6b). Considering the subset of these overlapping genes that also harbour carRNA m^6^A methylation, we identified enrichment of the TGF-β signalling and cytokine–cytokine receptor interaction-related pathways (Fig. 6b). Among them, the most representative gene is *TGFB1*, of which the promoter-associated RNAs are highly methylated (~67%) based on our m^6^A-SAC-seq^36^ quantification (Fig. 6c). The depletion of *RBFOX2* led to decreased m^6^A methylation, elevated RNA abundance and increased chromatin openness at the promoter region of *TGFB1* (Extended Data Fig. 10b). We also validated the increase in both *TGFB1* transcript level and TGFB1 protein level upon *RBFOX2* KD (Extended Data Fig. 10c,d). Moreover, the increase in *TGFB1* expression observed in *RBFOX2*-depleted cells could be reversed by overexpressing RBFOX2 in these cells (Extended Data Fig. 10e). Therefore, the RBFOX2-dependent *TGFB1* regulation is dependent on its paRNA m^6^A methylation.

Among the candidate genes (Extended Data Fig. 10f,g), the TGF-β signalling pathway stands out as playing a tumour suppressor role in haematologic malignancies and exhibits both antiproliferative and pro-differentiation effects^45^. To investigate whether this TGF-β signalling pathway regulation mediated through RBFOX2 also occurs in human AML cells, we first confirmed that *RBFOX2* KD caused a decrease in m^6^A level, an increase in paRNA abundance and a reduction in PRC2 binding at the promoter region of *TGFB1* (Extended Data Fig. 10h–j). These changes were associated with an increase of *TGFB1* at both transcript and protein levels (Extended Data Fig. 10k,l). In addition, we observed an upregulation of *TGFB1* expression in an AML PDX mouse model upon *RBFOX2* depletion (Extended Data Fig. 10m). We next treated NB4 cells with TGF-β activator and observed that it promoted myeloid differentiation, mimicking the effects of *RBFOX2* KD. Furthermore, TGF-β works in synergy with *RBFOX2* KD to promote myeloid differentiation (Extended Data Fig. 10n). Additionally, *TGFB1* depletion partially blocks myeloid differentiation induced by *RBFOX2* KD in human AML cells (Fig. 6d and Extended Data Fig. 10o,p). Taken together, our results confirm that suppression of tumourigenesis through *RBFOX2* depletion is dictated, at least partially, by the requirement of TGF-β for myeloid differentiation (Fig. 6e).

## Discussion

An emerging theme of RNA m^6^A methylation regulation is its role on carRNAs, which modulates chromatin state in mammals^1,24–27^ and plants^46^. However, how the RNA MTC is recruited to chromatin and how the specificity is achieved have not been well explored^23^. We currently lack knowledge on chromatin factors that could preferentially recognize m^6^A-modified RNAs and interface with the recruitment of the methyltransferase and downstream chromatin modifiers.

Known as a well-studied splicing factor, *Rbfox2* knockout causes heart failure in mice^47^. However, many cardiac phenotypes caused by the depletion of *Rbfox2* are not adequately explained by altered splicing^34^, indicating other functional pathways mediated through RBFOX2. Here we show that RBFOX2 functions as an m^6^A-binding protein and can regulate chromatin state through the RBM15/YTHDC1/PRC2 axis. RBFOX2 physically interacts with RBM15, a component of MTC, to mediate methylation of paRNAs. RBM15 also recruits YTHDC1, another m^6^A reader protein and chromatin modifier, to genomic sites occupied by RBFOX2 for chromatin silencing through the PRC2 complex. RBFOX2 itself can also preferentially bind to m^6^A-modified paRNA to further promote methylation and PRC2 recruitment to enhance transcription suppression.

YTHDC1 has been reported to regulate facultative chromatin marked with H3K9me3 through physically interacting with SETDB1 (refs. ^25,26,28^). We showed here that YTHDC1 interacts with the PRC2 complex to suppress chromatin accessibility in myeloid cells. When YTHDC1 is recruited to gene promoter regions, it not only destabilizes m^6^A-modified paRNA^1^, but also mediates H3K27 trimethylation to suppress transcription. This regulation on H3K27me3 by YTHDC1 is dependent on RBFOX2. Thus, our findings reveal the functional role of RBFOX2 as a m^6^A-binding protein on chromatin in promoting caRNA methylation for transcription suppression. This regulatory paradigm fills a critical missing link in the crosstalk between chromatins and m^6^A methylation of caRNAs.

## Methods

All experiments, including animal studies, were conducted in compliance with federal and state government guidelines and followed the approved protocol by the Institutional Animal Care and Use Committee (#17089) at City of Hope.

### Primary AML patient and healthy donor specimens

As previously reported in our publication^44^, human primary AML patient samples, as well as samples from healthy donors, were collected from bone marrow aspiration at City of Hope Hospital, Cincinnati Children’s Hospital or Dana-Farber/Harvard Cancer Center. This collection was carried out in accordance with the approved protocol by the institutional review board (#18147), and written informed consent was obtained from the participants at the time of diagnosis, relapse or remission.

Leukaemia blasts and mononuclear cells (MNCs) from human primary AML patient samples^44^ obtained from City of Hope Hospital and Cincinnati Children’s Hospital were separated using Ficoll-Paque density centrifugation and stored at −150 °C until needed. Leukaemia MNCs were grown in Iscove’s modified Dulbecco’s medium containing 20% foetal bovine serum (FBS), 1% penicillin–streptomycin, 2.5 µg ml^−1^ Plasmocin prophylactic and 10 ng ml^−1^ of rhSCF, rhTPO, rhFlt-3L, rhIL-3 and rhIL-6.

For the transduction process involving primary patient samples, six-well plates were coated with RetroNectin (T202, Takara) and kept at 4 °C overnight or for 2 h at room temperature. The viral supernatant was applied to the RetroNectin-coated plates, followed by centrifugation for 2 h at 2,000*g* and 32 °C. Primary AML patient cells were then placed onto the plates, and the samples were centrifuged for an additional 30 min at 600*g* and 32 °C. To achieve stable integration, infected cells underwent selection with 2 µg ml^−1^ puromycin (ant-pr-1, InvivoGen) for 2–4 days.

### Isolation, culture and transduction of CD34^+^ HSPCs

CD34^+^ HSPCs were extracted from UCB specimens, procured from StemCyte under the IRP protocol number 18147 approved by City of Hope. In brief, MNCs were separated from UCB using Ficoll-Paque density gradient centrifugation. The Human CD34 MicroBead Kit (130-046-702, Miltenyi Biotec) and MACS Separator were employed to enrich CD34^+^ cells from the MNCs, following the manufacturer’s guidelines. After enrichment, flow cytometry was conducted to evaluate the purity of the CD34^+^ cells. CD34^+^ HSPCs were then cultured in serum-free expansion medium, supplemented with rhTPO (10 ng ml^−1^), rhFlt-3L (10 ng ml^−1^), rhIL-3 (10 ng ml^−1^), rhIL-6 (10 ng ml^−1^) and rhSCF (100 ng ml^−1^).

### KD cell line construction

K562 was obtained from American Type Culture Collection (ATCC). Lentiviral amplification was performed by co-transfection of PCMV-DR8.2 dvpr, PCMV-VSV-G and short hairpin RNA (shRNA) in 293T cells (ATCC) by using Lipo 2000 (Thermo Fisher Scientific) according to the manufacturer’s instruction. Lentivirus was concentrated by precipitating the supernatant with 5× PEG-it (System Biosciences). K562 cells (ATCC) were transduced with indicated lentivirus in the presence of TransDux (System Biosciences) and treated with puromycin-containing medium (2 µg ml^−1^) to generate stable KD cell lines.

MonoMac-6, NOMO-1, MOLM13 and NB4 were obtained from Deutsche Sammlung von Mikroorganismen und Zellkulturen GmbH, Braunschweig, Germany. For mouse primary leukaemia cells, mouse MA9 cells were bone marrow cells collected from mice with MLL-AF9 leukaemia. Lenti-X 293T was purchased from Takara Bio. The TRC shRNAs targeting human *RBFOX2* (sh*RBFOX2*-1: TRCN0000294044; sh*RBFOX2*-2 TRCN0000311693) and mouse *Rbfox2* (sh*Rbfox2*-1: TRCN0000102342; sh*Rbfox2*-2: TRCN0000102343) as well as the non-targeting control shRNA were purchased from Sigma-Aldrich. Lentivirus particles for KD plasmids were all packaged with pMD2.G, psPAX2 (Addgene). Briefly, 5 μg pMD2.G, 5 μg psPAX2 and 5 μg construct for KD of specific genes were co-transfected into Lenti-X 293T cells in 100 mm cell culture dish with Effectene Transfection Reagent (301427, Qiagen). The virus particles were collected at 48 and 72 h after transfection and concentrated with PEG-it virus precipitation solution (LV810A-1, SBI). For infection, the concentrated virus or the viral supernatant was directly added into cells with presence of 4 µg ml^−1^ polybrene (H9268, Sigma-Aldrich) and then spinoculation was conducted at 32 °C, 1,200 rpm for 60 min. A total of 2 µg ml^−1^ puromycin (P8833, Sigma-Aldrich) was added to positively select transduced cells.

### Leukaemia cell culture

K562 cells were cultured in RPMI-1640 medium (Thermo Fisher Scientific supplemented with 10% FBS (Gibco), penicillin–streptomycin (Gibco) and 2 mM l-glutamine (Gibco) and grown at 37 °C with 5% CO_2_. *RBFOX2* stable KD K562 cells were cultured in the same medium as K562 cells containing 2 μg ml^−1^ puromycin and grown at 37 °C with 5% CO_2_.

For human leukaemia cells, NOMO-1, MOLM13 and NB4 were cultured in endotoxin-free RPMI-1640 supplemented with 10% FBS (Gemini Bio-Products), 1% HEPES (Gibco) and 1% penicillin–streptomycin (Gibco); MonoMac-6 cells were kept in RPMI-1640 with 10% FBS, 1% HEPES and 1% penicillin–streptomycin with the additions of 2 mM l-glutamine (Gibco), 1× MEM Non-Essential Amino Acid (Gibco), 1 mM sodium pyruvate (Gibco) and 9 μg ml^−1^ insulin (Gibco). Lenti-X 293T cells were grown in Dulbecco’s modified Eagle medium supplemented with 10% FBS and 1% penicillin–streptomycin. All the cells are not among commonly misidentified cell lines and were tested for mycoplasma contamination quarterly using a PCR Mycoplasma Detection Kit (Applied Biological Materials).

### Mouse housing and procedure

NRG-SGM3 (NRGS, RRID: IMSR_JAX:024099) mice were used for ‘human-in-mouse’ xenotransplantation model. The mice were originally purchased from the Jackson Laboratory and bred at the specific-pathogen-free core facilities of City of Hope according to standard procedures. For each experiment, similar number of male and female mice aged 6–8 weeks old were used and randomly allocated to each group. For xenograft mouse, 0.1 × 10^6^ MOLM13 cells were transplanted into NRGS recipient mice intravenously. The mice are housed under 12 h:12 h light–dark cycle. The room temperature is steadily kept at 22 ± 2 °C (71.6 ± 3.6 °F) with a consistent relative humidity level of around 50%. In addition to these, mice were provided with a clean, comfortable and enriching environment, including appropriate bedding and nesting materials following Institutional Animal Care and Use Committee regulations.

### Cell fractionation

K562 cells were fractionated according to the previously published procedure^53^. Briefly, 5 × 10^6^ to 1 × 10^7^ K562 cells were collected by centrifugation at 500*g* for 3 min, and washed once with 1 ml cold phosphate-buffered saline (PBS)/1 mM ethylenediaminetetraacetic acid (EDTA) buffer. The cell pellet was resuspended in 200 μl ice-cold lysis buffer (10 mM Tris–HCl, pH 7.5, 0.15% NP40 and 150 mM NaCl), and incubated on ice for 5 min. Then the cell lysate was gently pipetted up over 2.5 volumes of chilled sucrose cushion (24% RNase-free sucrose in lysis buffer), and centrifuged at 15,000*g* for 10 min at 4 °C. The supernatant was collected as cytoplasmic fraction. The nuclei pellet was gently rinsed once by adding 200 μl ice-cold PBS/1 mM EDTA without disturbing, and resuspended with 100 μl pre-chilled glycerol buffer (20 mM Tris–HCl, pH 7.9, 75 mM NaCl, 0.5 mM EDTA, 0.85 mM dithiothreitol, 0.125 mM phenylmethylsulfonyl fluoride and 50% glycerol) by gentle flicking of the tube. Next, 1.5 volumes of pre-chilled nuclei lysis buffer (10 mM HEPES, pH 7.6, 1 mM dithiothreitol, 7.5 mM MgCl_2_, 0.2 mM EDTA, 0.3 M NaCl, 1 M urea and 1% NP40) were added, followed by 2 min of vigorous vortexing. The sample was incubated on ice for 5 min and centrifuged at 4 °C with 15,000*g* for 2 min. All of the supernatant was collected as soluble nuclear fraction. The pellet was rinsed with cold PBS/1 mM EDTA, then collected as chromosome-associated fraction.

### RNA extraction

Total RNA was extracted from whole cells using TRIzol reagents (Thermo Fisher Scientific) following the manufacturer’s instructions.

To isolate the caRNA, the chromatin pellet was first obtained as described in the ‘Cell fractionation’ section. The pellet was then submerged in the TRIzol reagents and heated at 50 °C with shaking at 1,000 rpm for 1–2 h until the pellet completely dissolved. The caRNA was extracted according to the manufacturer’s instructions for TRIzol reagents.

To remove rRNA from whole cellular total RNA or caRNA, RiboMinus Eukaryote kit (Thermo Fisher Scientific) was used following the manufacturer’s protocol. For extraction of polyadenylated RNA from total RNA, the Dynabeads mRNA DIRECT kit (Thermo Fisher Scientific) was used according to the manufacturer’s instructions. The RNA concentration was measured using either NanoDrop 8000 Spectrophotometer (Thermo Fisher Scientific) or the Qubit Fluorometer (Thermo Fisher Scientific).

### RIP–LC–MS/MS

K562 cells were collected by centrifugation at 500*g* for 3 min. HepG2 cells were collected using a cell lifter (Corning), followed by centrifugation at 500*g* for 3 min. Cells were washed twice with cold Dulbecco’s PBS (DPBS). The cell pellet was resuspended in 1.5 ml of lysis buffer (50 mM Tris–HCl, pH 7.5, 150 mM NaCl, 1% NP40, 1:100 Protease Inhibitor Cocktail and 400 U ml^−1^ SUPERase In), pipetted up and down several times, and incubated on ice for 20 min. The cell lysate was then cleared by centrifugation at 15,000*g* for 15 min at 4 °C. Fifty microlitres of the supernatant was mixed with 1 ml of TRIzol reagents (Thermo Fisher Scientific) and saved as the input. The remaining supernatant was incubated with 10 μg RBFOX2 antibody-conjugated Protein G Dynabeads (Thermo Fisher Scientific) at 4 °C for 4 h with rotation. The sample was placed in a magnetic stand, and 50 μl of the supernatant was with 1 ml TRIzol reagents and saved as the unbound portion (flow-through, FT). The beads were washed five times with 1 ml of the ice-cold lysis buffer. Next, the beads were mixed with 1 ml TRIzol reagents, incubated at room temperature for 30 min and saved as the immunoprecipitated portion (IP). RNAs from all three portions (input, FT and IP) were recovered from TRIzol reagents according to the manufacturer’s instructions. RNAs were further purified by rRNA depletion using the RiboMinus Eukaryote Kit (Thermo Fisher Scientific) with size selection of RNA greater than 200 nt using RNA Clean & Concentrator Kits (Zymo Research) twice. m^6^A abundance was then quantified using LC–MS/MS.

### Quantitative analysis of m^6^A by LC–MS/MS

A total of 100 ng non-ribosomal RNA, polyadenylated RNA or RIP RNA was digested by 1 U nuclease P1 (Sigma) in 30 μl of buffer containing 20 mM NH_4_OAc for 2 h at 42 °C. Subsequently, 1× FastAP Buffer and 1 U FastAP Thermosensitive Alkaline Phosphatase (Thermo Fisher Scientific) were added, and the sample was incubated at 37 °C for an additional 2 h. The samples were then centrifuged through filters (0.22 μm pore size, 4 mm diameter, Millipore) and injected into a reverse-phase ultraperformance liquid chromatography (C18 column) coupled to Triple Quad 6500 System (AB SCIEX). Nucleosides were detected in positive electrospray ionization mode. The quantification of nucleosides was based on the nucleoside-to-base ion mass transitions of 282 to 150 for m^6^A, and 268 to 136 for A by using standard curves generated in the same batch of samples with pure nucleosides. The ratio of m^6^A to A was calculated on the basis of the calibrated concentrations. The quantification of nucleosides was based on the nucleoside-to-base ion mass transitions of 282 to 150 for m^6^A, and 268 to 136 for A by comparing with standard curves generated from pure nucleosides run in the same batch of samples. The ratio of m^6^A to A was calculated on the basis of the calibrated concentrations.

### Protein purification

BL21 competent *Escherichia coli* (NEB) was transformed with the plasmid pET–28a–MBP–RBFOX2 full length or pGEX–6P-1–RBFOX2 RRM, and cultured at 37 °C incubator. Cells were cooled in ice for 10 min when the optical density at 600 nm (OD_600_) reached 0.6, and isopropyl β-d-1-thiogalactopyranoside was added to a final concentration of 0.2 mM to induce protein expression. Cells were cultured at 16 °C with shaking at 200 rpm for an additional 20 h. Cells were collected by centrifugation and lysed in the buffer of 50 mM Tris–HCl (pH 7.5) and 300 mM NaCl with sonication at 10 s on/10 s off setting for 10 min at 4 °C. The soluble fraction was purified with HisTrap HP column (GE Healthcare) or GSTrap HP column (GE Healthcare) following the manufacturer’s instruction on an AKTA Purifier 10 system (GE Healthcare). Eluted protein was further purified by removing imidazole in the buffer with Amicon Ultra centrifugal filter units (Millipore). The purified protein was supplemented with 50% glycerol and stored at −80 °C for future use.

### Electrophoretic mobility shift assay

Carboxyfluorescein-labelled RNA probe was heated at 65 °C for 5 min and quickly placed on ice to denature the RNA probe. His–MBP–RBFOX2 full-length protein was diluted to concentration series of 100 nM, 200 nM, 400 nM, 500 nM, 600 nM and 2 μM in binding buffer (10 mM Tris–HCl 7.5, pH 7.5, 50 mM KCl, 5% glycerol and 1 U µl^−1^ Superase In). RNA probe (10 nM final concentration) and 2 µl protein (20 nM, 40 nM, 80 nM, 100 nM, 120 nM and 400 nM final concentration) were mixed in 10 μl binding buffer and incubated on ice for 30 min without disturbing. Eight microlitres of RNA–protein mixture was loaded to the gel (Novex 4 ~20% TBE gel) directly and run at 4 °C for 90 min at 90 V. Imaging was performed in Bio-Rad Molecular Imager FX.

### Oligo pulldown assay

One microgram biotinylated RNA probe was incubated with 10 µl pre-washed Dynabeads MyOne Streptavidin C1 (Thermo Fisher Scientific, 65001) at room temperature for 15 min with rotation and washed. RNA probe–beads conjugates were incubated with cleared K562 cell lysate or purified proteins in lysis buffer (50 mM Tris–HCl, pH 7.5, 150 mM NaCl, 1% NP40, 1:100 Protease Inhibitor Cocktail and 20 U ml^−1^ RNase inhibitor) at 4 °C for 2 h, and then washed five times with lysis buffer. Beads were boiled in 1× LDS loading buffer (Bio-Rad) at 95 °C for 10 min and analysed by western blot.

### Dot blot

The biotinylated RNA oligo solutions were diluted to a concentration of 2.5 ng µl^−1^, and 1 µl of the diluted RNA oligo solution was loaded onto an Amersham Hybond-N+ membrane (GE Healthcare). The membrane was air-dried and cross-linked twice by ultraviolet (UV) light at 120 mJ cm^−2^ using Stratalinker 2400. To block non-specific binding, the membrane was incubated with 5% fatty-acid-free bovine serum albumin (BSA) in PBST (PBS with 0.1% Tween-20) at room temperature for 1 h, followed by incubation with streptavidin–horseradish peroxidase (HRP) (Thermo Fisher Scientific) in PBST supplemented with 5% fatty-acid-free BSA at room temperature for another 1 h. The membrane was washed with PBST four times and imaged using SuperSignal West Dura Extended Duration Substrate kit (Thermo Fisher Scientific) on the FluroChem R machine (Proteinsimple).

### In vivo pulldown assay

K562 cells were lysed in lysis buffer (50 mM Tris–HCl, pH 7.5, 150 mM NaCl, 1% NP40, 1:100 Protease Inhibitor Cocktail and 20 U ml^−1^ RNase inhibitor) and incubated at 4 °C for 15 min. The lysate was cleared by centrifugation at 4 °C for 10 min at 15,000*g*. Twenty microlitres of the supernatant was kept as input portion. The remaining supernatant was incubated with 10 μg RBFOX2 antibody (Proteintech, cat. no. 12498-1-AP)-conjugated Protein G Dynabeads (Thermo Fisher Scientific) at 4 °C for 2 h with rotation. The aqueous phase was collected, recovered by TRIzol reagents according to manufacturer’s instruction, ethanol precipitated, dissolved in 15 µl water and saved as the FT portion. The beads were washed five times with lysis buffer. TRIzol reagent was added to beads and beads-bound RNA was purified following manufacturer’s protocol. The purified fraction was dissolved in 15 µl water and saved as RBFOX2-bound portion. m^6^A levels were measured by LC–MS/MS in each sample of input, FT and RBFOX2-bound.

### Nascent RNA transcription measured by qPCR

Six 6 cm plates of K562 cells were seeded at the same number of cells. 5-Ethynyl uridine was added to 0.5 mM at 60 min, 30 min and 10 min before collection. Total RNA was extracted by TRIzol reagents (Thermo Fisher Scientific) and nascent RNA was captured using the Click-iT Nascent RNA Capture Kit (Invitrogen) following the manufacturer’s protocols. Then 1:1,000 diluted m^6^A and non-m^6^A spike-in from the EpiMark *N*^6^-Methyladenosine Enrichment Kit (New England Biolabs) were added to total RNA proportionally. RNA quantities of interested RNAs were analysed by reverse-transcription quantitative polymerase chain reaction (qPCR).

### Co-IP

K562 cells were pelleted by centrifugation at 500*g* for 3 min and washed twice by PBS. The cell pellet was resuspended with cold lysis buffer (50 mM Tris–HCl, pH 7.5, 150 mM NaCl, 1% NP40, 1:100 Protease Inhibitor Cocktail and 20 U ml^−1^ RNase inhibitor), and incubated at 4 °C for 15 min with rotation. The lysate was centrifuged at 4 °C for 15 min at 15,000*g*. Fifty microlitres of the supernatant was saved as input. The rest of the supernatant was incubated with 1–2 μg of specific antibodies-conjugated or IgG-conjugated Protein G Dynabeads (Thermo Fisher Scientific) at 4 °C overnight with rotation. Beads were washed five times with the lysis buffer. Both beads and input samples were boiled in 1× LDS loading buffer (Bio-Rad) at 95 °C for 10 min and analysed by western blot.

### Western blot

The cell samples were lysed with RIPA buffer (Thermo Fisher Scientific) containing 1× protease inhibitor cocktail (Roche). The cell lysates were then mixed with 4× loading buffer (Bio-Rad) and boiled at 95 °C for 10 min. Denatured protein was cleared by centrifugation at 15,000*g* for 10 min at room temperature, loaded into 4–12% NuPAGE Bis-Tris gel (Thermo Fisher Scientific), and then transferred to polyvinylidene difluoride membrane. The membranes were blocked in 3% BSA (diluted in PBST) for 1 h at room temperature, incubated in a 1:1,000 diluted primary antibody solution at 4 °C overnight, washed with PBST and incubated in a 1:5,000 dilution of mouse monoclonal anti-rabbit IgG light chain (HRP) (Abcam, cat. no. ab37415) for 1 h at room temperature. Protein bands were detected by SuperSignal West Dura Extended Duration Substrate Kit (Thermo Fisher Scientific) on a FluroChem R (Proteinsimple). The primary antibodies used for western blot include rabbit polyclonal anti-RBFOX2 (Proteintech, cat. no. 12498-1-AP), rabbit polyclonal anti-GST tag (Cell Signaling Technology, cat. no. 2622), rabbit monoclonal anti-METTL3 (Abcam, cat. no. ab195352), rabbit polyclonal anti-METTL14 (Sigma-Aldrich, cat. no. HPA038002), rabbit polyclonal anti-RBM15 (Proteintech, cat. no. 10587-1-AP), rabbit polyclonal anti-YTHDC1 (Abcam, cat. no. ab122340), rabbit polyclonal anti-RBM15 (Proteintech, cat. no. 10587-1-AP). For His tag–MBP–RBFOX2 or GAPDH detection, the membranes were incubated with the 1:1,000 diluted mouse monoclonal anti-His tag (HRP conjugate) (Cell Signaling Technology, cat. no. 9991) or rabbit anti-GAPDH mAb (HRP conjugate) (Cell Signaling Technology, cat. no. 3683) respectively, instead of the incubation of the primary and secondary antibody.

### Protein interaction detected by PLA

K562 cells were collected by centrifugation at 500*g* for 3 min, and washed one time with DPBS. The cells were then suspended in and seeded in an eight-well chamber (Lab-Tek) with incubation for 10 min at room temperature. Next, cells were fixed with 4% paraformaldehyde by adding 1/3 volume of 16% paraformaldehyde and incubated at room temperature for 15 min, followed by permeabilization with 0.5% Trition X-100 in DPBS for additional 10 min at room temperature. Protein interactions were detected using the Duolink PLA Fluorescence system (Sigma) according to the manufacturer’s instructions. Briefly, K562 cells were blocked with Duolink Blocking Solution at 37 °C for 1 h, and then incubated with primary antibodies. The cells were then incubated with a mixture of Probe Anti-Rabbit PLUS reagent and Probe Anti-Mouse Minus reagent at 37 °C for 1 h. The ligation and amplification reactions were performed, and nuclei were stained with 1 mM Hoechst 33342 at room temperature for 10 min. Images were captured using Leica SP8 confocal microscope.

### IF imaging of RBFOX2 and YTHDC1

K562 cells were fixed and permeabilized as described in the ‘Protein interaction detected by PLA’ section. Then, cells were incubated with a 1:200 diluted primary antibodies of RBFOX2 (Abcam, mouse antibody, cat. no. ab57154) and a 1:200 diluted YTHDC1 (Abcam, rabbit antibody, cat. no. ab122340) at room temperature for 1 h, followed by incubation with a 1:1,000 diluted mixture of goat anti-mouse IgG (H + L) cross-adsorbed secondary antibody, Alexa Fluor 647 (Thermo Fisher Scientific) and goat anti-rabbit IgG (H + L) cross-adsorbed secondary antibody, Alexa Fluor 568 (Thermo Fisher Scientific) at room temperature for an additional hour. Nuclei were stained with 1 mM Hoechst 33342 for 10 min at room temperature. Images were captured using Leica SP8 confocal microscope and analysed by ImageJ software.

### Enzyme-linked immunosorbent assay for TGF-β 1 (TGFB1)

K562 cells were seeded at a density of 0.3 × 10^6^ cells ml^−1^ in completed culture medium and incubated at 37 °C with 5% CO_2_. The supernatant was collected by centrifugation at 500*g* for 3 min at 24 h (day 1), 48 h (day 2) and 72 h (day 3). The concentration of TGF-β was measured by using Human TGF**-**β 1 ELISA Kit (Abcam, cat. no. ab100647) following the manufacturer’s protocol.

### Cell proliferation/growth and apoptosis assays

The cell proliferation/growth was assessed by non-radioactive cell proliferation assay (MTT, G4100, Promega) following the manufacturer’s instructions. Briefly, cells were seeded into 96-well plate in triplicates at the density of 10,000 cells per 100 μl. Dye solution was added at indicated timepoints and incubated at 37 °C for 3–4 h before adding of solubilization/stop to stop the reaction. The absorbance at 570 nm (with reference at 630 nm) was read on the next day. For apoptosis assays, APC Annexin V Apoptosis Detection Kit (eBiosciences) was used following the manufacturer’s manuals.

### Cell differentiation assay

After counting with trypan blue exclusion, 20,000–50,000 cells were loaded and cytospins were prepared at 1000 rpm for 5 min. Slides were air-dried and stained with StainRITE Wright-Giemsa Stain Solution (Polysciences) and mounted with Poly-mount (Polysciences). For flow cytometric analysis, K562, NB4 or MOLM13 cells with different treatments were collected and washed with chilled PBS, followed by staining with APC-CD61 (eBioscience, cat. no. 17-0619-42), PE-labelled anti-CD11b (BioLegend, cat. no. 101208) and APC-labelled anti-CD14 (eBioscience, cat. no. 17-0149-41) antibodies for 25 min. Cells were then fixed and analysed on a BD LSRFortessa or FACSAria III analyser (BD Biosciences).

### Colony-forming assay

For colony-forming assays using human leukaemia cells, the transduced cells were seeded into MethoCult H4434 Classic medium (StemCell Technologies) with the addition of 2.5 µg ml^−1^ puromycin. Cultures were incubated at 37 °C in a humidified atmosphere of 5% CO_2_ for 10 days before counting.

### In vitro LDAs

Bone marrow cells collected from MLL-AF9 leukaemia mice were stained with PE-CD45.2, sorted on a BD FACSAria III cell sorter (BD Biosciences) and transduced with shRNAs targeting mouse *Rbfox2*. The transduced cells were plated into ColonyGEL methylcellulose medium with 10 ng ml^−1^ of murine recombinant IL-3, IL-6 and GM-CSF and 30 ng ml^−1^ of murine recombinant SCF, along with 2.5 μg ml^−1^ of puromycin (Sigma-Aldrich). Seven days later, the colony cells were collected and plated into 48-well plates with six different doses of donor cells for each group. The number of wells that developed MA9 clones was counted. ELDA^54^ software was used to estimate the frequency of LSCs/LICs.

### Chromosome-associated RNA (caRNA) m^6^A MeRIP-seq

caRNA was isolated from the chromosome-associated fraction of K562 cells. Non-ribosomal RNA was purified from total RNA by RiboMinus Eukaryote kit (Thermo Fisher Scientific). m^6^A immunoprecipitation (IP) was performed using EpiMark *N*^6^-Methyladenosine Enrichment Kit (New England Biolabs) following the manufacturer’s protocols. Libraries were prepared with SMARTer Stranded Total RNA-Seq Kit v2 (Takara) according to the manufacturer’s protocols.

### ChIP–seq

K562 cells were cross-linked by adding 1% formaldehyde directly to the medium and slowly shaking at room temperature for 8 min for histone modifications or 15 min for TFs. Cross-linking was stopped by adding glycine to a final concentration of 0.125 M and incubating for 5 min at room temperature with slow shaking. The cells were washed twice with ice-cold PBS. Chromatin IP was performed by the iDeal ChIP–seq Kit for Histone Marks (Diagenode) or the iDeal ChIP-seq kit for Transcription Factors (Diagenode) following the manufacturer’s protocols. Library preparation was performed with KAPA HyperPrep Kit for NGS DNA Library Prep (Roche) according to the manufacturer’s protocols.

### KAS-seq

Kethoxal-assisted single-stranded DNA sequencing (KAS-seq) was performed following the previously published procedure^55^. Briefly, K562 cells were incubated in completed culture medium containing 5 mM *N*_3_-kethoxal for 5 min at 37 °C with 5% CO_2_. Cells were collected by centrifugation and washed twice with DPBS. The genomic DNA (gDNA) was isolated using PureLink genomic DNA mini kit (Thermo Fisher Scientific, K182002), and the gDNA concentration was measured by NanoDrop 8000 Spectrophotometer (Thermo Fisher Scientific). Two micrograms gDNA was diluted in 100 µl reaction buffer of 1× DPBS, 25 mM K_3_BO_3_ and 1 mM DBCO–PEG_4_–biotin (Sigma, 760749). The reaction was performed at 37 °C for 1 h with gentle shaking. Next, 5 µl RNase A (Thermo Fisher Scientific, 12091039) was added into the mixture followed by incubation at 37 °C for 15 min. Labelled gDNA was purified with DNA Clean & Concentrator-5 kit (Zymo Research, D4013) and resuspended into 100 µl buffer of 25 mM K_3_BO_3_. One microgram gDNA was fragmented to 150–350 bp size using Bioruptor Pico at 30 s on/30 s off setting for 30 cycles. Five per cent of the fragmented DNA was saved as input, and the remaining 95% was used for biotinylated DNA enrichment with 10 µl pre-washed Dynabeads MyOne Streptavidin C1 (Thermo Fisher Scientific, 65001). The beads were washed and heated in 15 µl H_2_O at 95 °C for 10 min to elute bound DNA. Eluted DNA and its corresponding input were used for library preparation by using Accel-NGS Methyl-seq DNA library kit (Swift, 30024).

### m^6^A-SAC-seq

m^6^A-SAC-seq was performed following the previously published procedure^36^. Briefly, 50 ng of K562 caRNA and RBFOX2-bound RNA were fragmented with RNA Fragmentation Reagents (Thermo Fisher Scientific, AM8740) at 70 °C for 5 min, followed by end repair with T4 Polynucleotide Kinase (New England Biolabs, M0201) at 37 °C for 1 h. Next, spike-in mix was added, and ligation of RNA 3′ biotinylated adaptor was performed by using T4 RNA ligase 2, truncated KQ (New England Biolabs, M0373). Excess adaptors were digested with 5′ deadenylase (New England Biolabs) and RecJ (New England Biolabs). RNAs were enriched by Dynabead MyOn Streptavidin C1 beads (Thermo Fisher Scientific, 65001) following the manufacturer’s instructions, and labelled by Mjdim1 with allyl-SAM for two rounds at 50 °C for 1 h, followed by iodine labelling at room temperature for another 1 h. Reverse transcription was carried out with recombinant HIV reverse transcriptase (Worthington Biochemical, LS05006) at 37 °C for 2 h, and template RNAs were digested with RNase H (New England Biolabs). The libraries were prepared by complementary DNA adaptor ligation with T4 RNA ligase 1, high concentration (New England Biolabs) and PCR amplification with NEBNext Multiplex Oligos for Illumina (New England Biolabs).

### CLIP-seq

CLIP-seq experiments were conducted with slight modifications based on a published protocol^56^. Briefly, K562 cells were cross-linked twice on ice with UV irradiation (254 nm) at 150 mJ cm^−2^ using Stratalinker 2400. Cells were then collected by centrifugation, lysed and treated with 4 U ml^−1^ RNase I (Thermo Fisher Scientific) and 4 U ml^−1^ Turbo DNase (Thermo Fisher Scientific) at 37 °C for 5 min. Next, the cell lysate was cleared by centrifugation at 15,000*g*, 4 °C for 15 min, and kept on ice for the subsequent IP. RBFOX2 antibodies (Proteintech, cat. no. 12498-1-AP) were pre-conjugated to Protein A/G Magnetic Beads (Thermo Fisher Scientific) by incubation at room temperature for 45 min. The cell lysate was then added to the pre-conjugated beads and incubated at 4 °C overnight. RBFOX2-bound RNAs were end-repaired with T4 PNK (Thermo Fisher Scientific), released by proteinase K (Thermo Fisher Scientific) treatment, and recovered using Oligo Clean & Concentrator Kits (Zymo Research). RNA libraries were prepared using NEBNext Small RNA Library Prep Set (NEB) according to the manufacturer’s protocol.

### RNA extraction and reverse-transcription qPCR analysis

Total RNA was isolated using the miRNeasy mini kit (Qiagen) according to the manufacturer’s instructions and quantified by UV spectrophotometry. For analysis of mRNA expression, 200–500 ng of RNA was reverse-transcribed into cDNA in a total reaction volume of 10 μl with the QuantiTect Reverse Transcription Kit (Qiagen). Quantitative real-time PCR analysis was then performed with 0.5 μl diluted cDNA (with 2.5-fold dilution) using Maxima SYBR green qPCR master mix (Thermo Fisher Scientific) on the QuantStudio 7 Flex PCR system (Thermo Fisher Scientific). GAPDH or ACTB was used as endogenous control.

### RNA sequencing data analysis

Raw reads were trimmed with Trimmomatic-0.39 (ref. ^57^), then aligned to human genome and transcriptome (hg38) using HISAT (version 2.1.0) (ref. ^58^) with ‘–rna-strandness RF’ parameters. Annotation files (version v29, 2018-08-30, in gtf format) were downloaded from GENCODE database (https://www.gencodegenes.org/)^59^.

For caRNA m^6^A MeRIP-seq, mapped reads were separated by strands with samtools (version 1.9) (ref. ^60^) and m^6^A peaks on each strand were called using MACS (version 2) (ref. ^61^) with parameter ‘-nomodel,–keep-dup 5, -g 1.3e8, -extsize 150’ separately. Significant peaks with *q* < 0.01 identified by MACS2 (ref. ^61^) were considered. Peaks identified in at least two biological replicates were merged using bedtools (v.2.26.0) (ref. ^60^) and were used in the following analysis.

Reads from input of m^6^A MeRIP-seq were counted for each GENCODE^59^ annotated gene using HTSeq^62^ and then differentially expressed genes were called using DESeq2 (ref. ^63^) package in R requiring at least ten read counts in at least two samples with adjusted *P* value <0.05.

### m^6^A-SAC-seq data analysis

SAC-seq data analysis was preformed following the previously published procedure^36^ with modifications. Briefly, raw reads containing adapter sequence were clipped using Cutadapt v2.10 (ref. ^64^). Unique molecular identifiers were used for accurate detection and removal of PCR duplicates by using ‘clumpify.sh’ from BBMAP tools (https://sourceforge.net/projects/bbmap/)^65^. The pre-processed reads were then aligned to human genome and transcriptome (hg38) using STAR v2.7.3a (ref. ^66^) with parameters according to the ENCODE long RNA-seq processing pipeline, except for allowing mismatches depending on read length to capture more potential base mutations (–outFilterMismatchNoverReadLmax 0.06). After mapping, mutation calling was performed using VarScan v2.3.9 (ref. ^67^) subcommand ‘somatic’ in two pair-wise comparisons. Finally, sites for which (1) the reference position was adenine (A); (2) *P* value <0.05; (3) read converge >5, converge of reads with mutation at A >3 and mutation frequency >5%; (4) mutation frequency difference compared MjDim1 enzymes-treated sample with untreated one >5% were kept for downstream analysis.

### CLIP-seq data analysis

Low-quality reads were filtered using ‘fastq_quality_filter’, and adapters were clipped using ‘fastx_clipper’, then adapter-free was collapsed to remove PCR duplicates by using ‘fastx_collapser’, and finally reads longer than 15 nt were retained for further analysis (http://hannonlab.cshl.edu/fastx_toolkit/). Reads from rRNA were removed. The pre-processed reads were mapped to hg38 using bowtie^68^ with ‘-v 3 -m 10 -k 1 –best –strata’ parameters.

### DNA sequencing data analysis

Raw reads were trimmed with Trimmomatic-0.39 (ref. ^57^) and then mapped to human genome (hg38) using bowtie2 (version 2.4.1) (ref. ^69^) with default mode, where multiple alignments are searched and the best one is reported.

For ChIP–seq, peaks were called using HOMER^48^ in histone (for histone modification ChIP–seq) or factor (for transcriptional factor ChIP–seq) mode.

For KAS-seq, elongation rate was calculated as number of reads per kilobase per million reads in the interval [transcription start site (TSS) + 500, transcription termination site (TTS)] divided by reads per kilobase per million reads in the interval [TSS-50, TSS + 250] (ref. ^70^).

### Matrix build and quantification for peak co-localization

Histone modification and transcriptional factor binding sites in format of BED files were downloaded from the ENCODE project^30^. All binding sites within each cell line (K562 or HepG2), together with the corresponding m^6^A peaks on chromatin-associated RNAs, were pooled and served as reference peaks for integrative analysis. Later, a binary matrix was built with reference peaks as rows, histone modification/transcriptional factor/m^6^A peaks as columns, and 0/1 as entries, where ‘1’ indicates a target peak overlapped with the reference peak, while ‘0’ indicates non-overlap between them. Finally, association index^71^ was calculated to quantify the co-localization between histone modification/transcriptional factor with m^6^A peaks.

### Enrichment analysis

Functional enrichment analysis was performed with DAVID^72^ using default parameters.

### Visualization

Cytoscape^73^ was used for network analysis and visualization.

### Statistics and reproducibility

At least three biological replicates were used in each experiment unless otherwise stated. Data were presented as the mean ± standard error of the mean (s.e.m.) or standard deviation (s.d.). Two-tailed, unless otherwise stated, Student’s *t*-test, non-parametric Wilcoxon–Mann–Whitney test (Wilcoxon rank-sum test, two-sided) or analysis of variance (ANOVA) (Dunnett’s multiple comparisons test) were performed to assess the statistical significance between groups and *P* value was indicated in the figure legends. Pearson correlation coefficient (PCC) was calculated to assess correlation. To evaluate the scatter of the data points around the fitted regression line, *R*^2^ was used to indicate the percentage of the dependent variable variation that a linear model explains. For box plots, the centre line represents the median, the box limits show the upper and lower quartiles, and whiskers represent 1.5× the interquartile range. No statistical methods were used to pre-determine sample sizes, but our sample sizes were similar to those reported in previous publications^1,74^. Samples were located into experimental group randomly. All the western blotting, PLA and IF experiments were repeated at least two biologically independent times with similar results.

### Reporting summary

Further information on research design is available in the Nature Portfolio Reporting Summary linked to this article.

## Online content

Any methods, additional references, Nature Portfolio reporting summaries, source data, extended data, supplementary information, acknowledgements, peer review information; details of author contributions and competing interests; and statements of data and code availability are available at 10.1038/s41556-023-01213-w.

## Supplementary information

Supplementary Information Supplementary Fig. 1. Unstained cells were used to set the gates for single staining and dual staining analysis. In brief, all the events except debris were gated for singlets analysis, then 4′,6-diamidino-2-phenylindole-negative live cells were gated for fluorescence analysis in which unstained control cells were used to set the gate for positive populations.

Reporting Summary

## Data Availability

Sequencing data that support the findings of this study have been deposited in the Gene Expression Omnibus (GEO) under accession code GSE205714. Previously published six datasets that were re-analysed here are available under accession codes GSE105870, GSE106042, GSE175263, GSE88446, GSE80913 and GSE88200. Source data are provided with this paper. All other data supporting the findings of this study are available from the corresponding author on reasonable request.

## Source data

Source Data Fig. 1 Unprocessed western blots.
Source Data Fig. 1 Statistical source data.
Source Data Fig. 2 Unprocessed western blots.
Source Data Fig. 3 Unprocessed western blots.
Source Data Fig. 4 Statistical source data.
Source Data Fig. 5 Statistical source data.
Source Data Fig. 6 Statistical source data.
Source Data Extended Data Fig. 2 Unprocessed western blots.
Source Data Extended Data Fig. 2 Statistical source data.
Source Data Extended Data Fig. 3 Unprocessed western blots.
Source Data Extended Data Fig. 4 Unprocessed western blots.
Source Data Extended Data Fig. 5 Unprocessed western blots.
Source Data Extended Data Fig. 5 Statistical source data.
Source Data Extended Data Fig. 6 Unprocessed western blots.
Source Data Extended Data Fig. 6 Statistical source data.
Source Data Extended Data Fig. 7 Unprocessed western blots.
Source Data Extended Data Fig. 7 Statistical source data.
Source Data Extended Data Fig. 9 Unprocessed western blots.
Source Data Extended Data Fig. 10 Unprocessed western blots.
Source Data Extended Data Fig. 10 Statistical source data.
