# Supplementary material for: RBFOX2 recognizes N6-methyladenosine to suppress transcription and block myeloid leukaemia differentiation
Source: Nat Cell Biol. 2023 Aug 28;25(9):1359–68. doi: 10.1038/s41556-023-01213-w (PMC10495261; doi:10.1038/s41556-023-01213-w)
Supplement: Supplementary file 1 — Supplementary Fig. 1. Unstained cells were used to set the gates for single staining and dual staining analysis. In brief, all the events except debris were gated for singlets analysis, then 4′,6-diamidino-2-phenylindole-negative live cells were gated for fluorescence analysis in which unstained control cells were used to set the gate for positive populations. [file 41556_2023_1213_MOESM1_ESM.pdf]

nature cell biology

Article https://doi.org/10.1038/s41556-023-01213-w

RBFOX2 recognizes N^6-methyladenosine to suppress transcription and block myeloid leukaemia differentiation

In the format provided by the authors and unedited

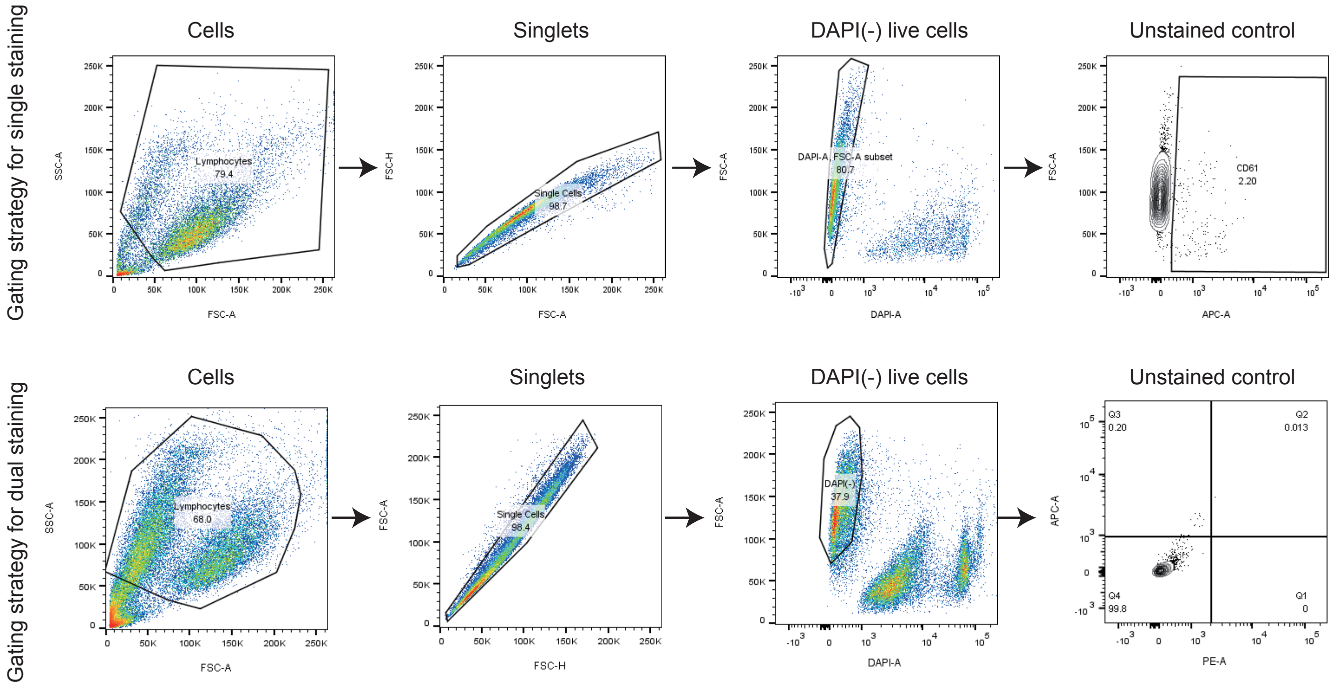

Supplementary Fig. 1: Unstained cells were used to set the gates for single staining and dual staining analysis. In brief, all the events except debris were gated for singlets analysis, then DAPI negative live cells were gated for fluorescence analysis in which unstained control cells were used to set the gate for positive populations.
