# Supplementary material for: RBFOX2 recognizes N6-methyladenosine to suppress transcription and block myeloid leukaemia differentiation
Source: Nat Cell Biol. 2023 Aug 28;25(9):1359–68. doi: 10.1038/s41556-023-01213-w (PMC10495261; doi:10.1038/s41556-023-01213-w)
Supplement: Supplementary file 2 — Reporting Summary [file 41556_2023_1213_MOESM2_ESM.pdf]

nature portfolio

Corresponding author(s): Chuan He

Last updated by author(s): Jul 20, 2023

Reporting Summary

Nature Portfolio wishes to improve the reproducibility of the work that we publish. This form provides structure for consistency and transparency in reporting. For further information on Nature Portfolio policies, see our Editorial Policies and the Editorial Policy Checklist.

Statistics

For all statistical analyses, confirm that the following items are present in the figure legend, table legend, main text, or Methods section.

n/a	Confirmed	
☐	☒	The exact sample size (*n*) for each experimental group/condition, given as a discrete number and unit of measurement
☐	☒	A statement on whether measurements were taken from distinct samples or whether the same sample was measured repeatedly
☐	☒	The statistical test(s) used AND whether they are one- or two-sided<br>*Only common tests should be described solely by name; describe more complex techniques in the Methods section.*
☐	☒	A description of all covariates tested
☐	☒	A description of any assumptions or corrections, such as tests of normality and adjustment for multiple comparisons
☐	☒	A full description of the statistical parameters including central tendency (e.g. means) or other basic estimates (e.g. regression coefficient) AND variation (e.g. standard deviation) or associated estimates of uncertainty (e.g. confidence intervals)
☐	☒	For null hypothesis testing, the test statistic (e.g. *F*, *t*, *r*) with confidence intervals, effect sizes, degrees of freedom and *P* value noted<br>*Give P values as exact values whenever suitable.*
☒	☐	For Bayesian analysis, information on the choice of priors and Markov chain Monte Carlo settings
☒	☐	For hierarchical and complex designs, identification of the appropriate level for tests and full reporting of outcomes
☐	☒	Estimates of effect sizes (e.g. Cohen's *d*, Pearson's *r*), indicating how they were calculated

Our web collection on statistics for biologists contains articles on many of the points above.

Software and code

Policy information about availability of computer code

Data collection	*Provide a description of all commercial, open source and custom code used to collect the data in this study, specifying the version used OR state that no software was used.*
Data analysis	Trimmomatic (version 0.39)<br>FastQC (version 0.11.8)<br>Flowjo (version 10)<br>Cutadapt (version 2.10)<br>BBMap (version 38.86)<br>Hisat2 (version 2.1.0)<br>Bowtie (version 1.0.0)<br>Bowtie2 (version 2.4.1)<br>STAR (version 2.7.3a)<br>Samtools (version 1.9)<br>HTSeq (version 0.12.4)<br>DESeq2 (version 1.26.0)<br>Homer (version 4.11)<br>MACS2 (version 2.2.7.1)<br>Bedtools (version 2.26)<br>VarScan2 (version 2.3.9)<br>DAVID 6.8 (Oct. 2016)<br>Cytoscape (version 3.7.2)

ImageJ (version 1.52p)
fastx_toolkit (http://hannonlab.cshl.edu/fastx_toolkit/)

For manuscripts utilizing custom algorithms or software that are central to the research but not yet described in published literature, software must be made available to editors and reviewers. We strongly encourage code deposition in a community repository (e.g. GitHub). See the Nature Portfolio guidelines for submitting code & software for further information.

Data

Policy information about availability of data

All manuscripts must include a data availability statement. This statement should provide the following information, where applicable:

- Accession codes, unique identifiers, or web links for publicly available datasets
- A description of any restrictions on data availability
- For clinical datasets or third party data, please ensure that the statement adheres to our policy

Sequencing data that support the findings of this study have been deposited in the Gene Expression Omnibus (GEO) under accession code GSE205714. Previously published six data that were re-analysed here are available under accession code GSE105870, GSE106042, GSE175263, GSE88446, GSE80913, GSE88200. Source data are provided with this study. All other data supporting the findings of this study are available from the corresponding author on reasonable request.

Human research participants

Policy information about studies involving human research participants and Sex and Gender in Research.

Reporting on sex and gender	Has been reported in Han, Li et al., Cell Stem Cell, 2023 (PMID: 36608679)
Population characteristics	Has been reported in Han, Li et al., Cell Stem Cell, 2023 (PMID: 36608679)
Recruitment	Has been reported in Han, Li et al., Cell Stem Cell, 2023(PMID: 36608679)
Ethics oversight	As previously reported in our publication (Han, Li et al., Cell Stem Cell, 2023, PMID: 36608679), human primary AML patient samples, as well as samples from healthy donors, were collected from bone marrow aspiration at City of Hope Hospital, Cincinnati Children's Hospital, or Dana-Farber/ Harvard Cancer Center. This collection was carried out in accordance with the approved protocol by the institutional review board (IRB, #18147), and written informed consent was obtained from the participants at the time of diagnosis, relapse, or remission.

Note that full information on the approval of the study protocol must also be provided in the manuscript.

Field-specific reporting

Please select the one below that is the best fit for your research. If you are not sure, read the appropriate sections before making your selection.

☒ Life sciences ☐ Behavioural & social sciences ☐ Ecological, evolutionary & environmental sciences

For a reference copy of the document with all sections, see nature.com/documents/nr-reporting-summary-flat.pdf

Life sciences study design

All studies must disclose on these points even when the disclosure is negative.

Sample size	No statistical methods were used to pre-determine sample sizes but our sample sizes are similar to those reported in our previous publications (Liu et al., Science, 2020, PMID: 31949099 and Shen et al., Cell Stem Cell, 2020, PMID: 32402250)
Data exclusions	No data were excluded from the analysis.
Replication	The experiments were repeated a minimum of three times, unless stated otherwise.
Randomization	Samples were allocated into experimental groups randomly.
Blinding	The investigators were blinded to group allocation during data collection and analysis.

Reporting for specific materials, systems and methods

We require information from authors about some types of materials, experimental systems and methods used in many studies. Here, indicate whether each material, system or method listed is relevant to your study. If you are not sure if a list item applies to your research, read the appropriate section before selecting a response.

March 2021

Materials & experimental systems

n/a	Involved in the study
☐	☒ Antibodies
☐	☒ Eukaryotic cell lines
☒	☐ Palaeontology and archaeology
☐	☒ Animals and other organisms
☒	☐ Clinical data
☒	☐ Dual use research of concern

Methods

n/a	Involved in the study
☐	☒ ChIP-seq
☐	☒ Flow cytometry
☒	☐ MRI-based neuroimaging

Antibodies

Antibodies used

Rabbit polyclonal anti-RBFOX2; Proteintech; Cat#12498-1-AP
Mouse monoclonal anti-RBFOX2; Abcam; Cat. #ab57154
Rabbit polyclonal anti-RBM15; Proteintech; Cat#10587-1-AP
Rabbit polyclonal anti-YTHDC1; Abcam; Cat#ab122340
Rabbit polyclonal anti-METTL14; Sigma-Aldrich; Cat#HPA038002
Rabbit monoclonal anti-METTL3; Abcam; Cat#ab195352; Clone#EPR18810
Rabbit polyclonal anti-EZH2; Cell Signaling Technology; Cat#5246; Clone#D2C9
Rabbit monoclonal anti-SUZ12; Cell Signaling Technology; Cat#3737; Clone# D39F6
HRP Goat Anti-Rabbit IgG (H+L); Cell Signaling Technology; Cat#7074
Mouse monoclonal Anti-Rabbit IgG light chain (HRP); Abcam; Cat#ab99697; Clone#SB62a
Rabbit IgG; Abcam; Cat#ab37415
Rabbit polyclonal anti-GST tag; Cell Signaling Technology; Cat#2622
Mouse monoclonal anti-his tag (HRP conjugate); Cell Signaling Technology; Cat#9991; Clone#27E8
Rabbit anti-H3K27me3; Cell Signaling Technology; Cat#9733; Clone# C36B11
Rabbit anti-H3K4me3; Cell Signaling Technology; Cat#9751; Clone# C42D8
Goat anti-Mouse IgG (H+L) Cross-Adsorbed Secondary Antibody, Alexa Fluor™ 647; Thermo Fisher Scientific; Cat#A-21235
Goat anti-Rabbit IgG (H+L) Cross-Adsorbed Secondary Antibody, Alexa Fluor™ 568; Thermo Fisher Scientific; Cat#A-11011
Rabbit anti-GAPDH (14C10) mAb (HRP Conjugate); Cell Signaling Technology; Cat#3683
Anti-Human CD11b, PE; BioLegend; Cat#101208
Anti-Human CD14, APC; eBioscience; Cat#17-0149-41
Anti-Human CD61, APC; eBioscience; Cat#17-0619-42

Validation

Commercial antibodies were validated by the manufacturer (see below link).
RBFOX2 (https://www.ptglab.com/products/RBM9-Antibody-12498-1-AP.htm)
RBM15 (https://www.ptglab.com/products/RBM15-Antibody-10587-1-AP.htm)
YTHDC1 (https://www.abcam.com/products/primary-antibodies/ythdc1-antibody-ab122340.html)
METTL14 (https://www.sigmaaldrich.com/US/en/product/sigma/hpa038002)
METTL3 (https://www.abcam.com/products/primary-antibodies/mettl3-antibody-epr18810-ab195352.html)
EZH2 (https://www.cellsignal.com/products/primary-antibodies/ezh2-d2c9-xp-rabbit-mab/5246)
SUZ12 (https://www.cellsignal.com/products/primary-antibodies/suz12-d39f6-xp-rabbit-mab/3737)
HRP Goat Anti-Rabbit IgG (H+L) (https://www.abcam.com/products/secondary-antibodies/rabbit-mouse-igg-hl-biotin-preadsorbed-ab7074.html)
Mouse monoclonal Anti-Rabbit IgG light chain (HRP) (https://www.abcam.com/products/secondary-antibodies/mouse-monoclonal-sb62a-rabbit-igg-light-chain-hrp-ab99697.html)
Rabbit IgG (https://www.abcam.com/products/primary-antibodies/rabbit-igg-polyclonal-isotype-control-ab37415.html)
Rabbit polyclonal anti-GST tag (https://www.cellsignal.com/products/primary-antibodies/gst-tag-antibody/2622)
Mouse monoclonal anti-his tag (HRP conjugate) (https://www.cellsignal.com/products/antibody-conjugates/his-tag-27e8-mouse-mab-hrp-conjugate/9991)
Rabbit anti-H3K27me3 (https://www.cellsignal.com/products/primary-antibodies/tri-methyl-histone-h3-lys27-c36b11-rabbit-mab/9733)
Rabbit anti-H3K4me3 (https://www.cellsignal.com/products/primary-antibodies/tri-methyl-histone-h3-lys4-c42d8-rabbit-mab/9751)
Goat anti-Mouse IgG (H+L) Cross-Adsorbed Secondary Antibody (https://www.thermofisher.com/antibody/product/Goat-anti-Mouse-IgG-H-L-Cross-Adsorbed-Secondary-Antibody-Polyclonal/A-21235)
Goat anti-Rabbit IgG (H+L) Cross-Adsorbed Secondary Antibody (https://www.thermofisher.com/antibody/product/Goat-anti-Rabbit-IgG-H-L-Cross-Adsorbed-Secondary-Antibody-Polyclonal/A-11011)
Rabbit anti-GAPDH (14C10) mAb (HRP Conjugate) (https://www.cellsignal.com/products/antibody-conjugates/gapdh-14c10-rabbit-mab-hrp-conjugate/3683)
Anti-Human CD11b, PE (https://www.biolegend.com/en-gb/antibodies-and-more/pe-anti-mouse-human-cd11b-antibody-349)
Anti-Human CD14, APC (https://www.labome.com/product/Invitrogen/11-0149-41.html)
Anti-Human CD14, APC (https://www.thermofisher.com/antibody/product/CD61-Integrin-beta-3-Antibody-clone-VI-PL2-Monoclonal/17-0619-42)

Eukaryotic cell lines

Policy information about cell lines and Sex and Gender in Research

Cell line source(s)

K562 was obtained from America Type Culture Collection (ATCC).
Mono-mac-6, NOMO-1, MOLM13 and NB4 were obtained from DSMZ.
Lenti-X 293T was bought from Takara.

	CD34+ HSPCs: We purchased the cord blood from StemCyte company with IRB protocol number 18147. We isolated the CD34+ HSPCs from the cord blood in the lab using CD34 MicroBead Kit, human (130-046-702, Miltenyi Biotech)
Authentication	The cell lines used were authenticated.
Mycoplasma contamination	All cell lines were tested negative for Mycoplasma contamination
Commonly misidentified lines (See ICLAC register)	No misidentified cell lines were used in this study.

Animals and other research organisms

Policy information about studies involving animals; ARRIVE guidelines recommended for reporting animal research, and Sex and Gender in Research

Laboratory animals	NRG-SGM3 (NRGS, RRID: IMSR_JAX:024099) mice from Jackson laboratory.<br>For each experiment, 6- to 8-week-old mice were used and randomly allocated to each group.
Wild animals	No wild animals were used
Reporting on sex	Similar number of male and female mice aged 6-8 weeks were used in the human AML cells xenograft studies. Based on our knowledge, sex and leukemogenesis of human AML cells in NRGS mice are not relevant. We didn't observe significant difference in leukemogenesis and survival between male and female mice.
Field-collected samples	The mice were bred at the specific-pathogen-free core facilities of City of Hope according to standard procedures. All animal studies listed below were conducted in accordance with federal and state government guidelines and IACUC protocols (#17089) approved by City of Hope. No field collected samples were used in the study.
Ethics oversight	All animal studies were conducted in accordance with federal and state government guidelines and IACUC protocols (#17089) approved by City of Hope

Note that full information on the approval of the study protocol must also be provided in the manuscript.

ChIP-seq

Data deposition

☒ Confirm that both raw and final processed data have been deposited in a public database such as GEO.

☒ Confirm that you have deposited or provided access to graph files (e.g. BED files) for the called peaks.

Data access links<br>*May remain private before publication.*	https://www.ncbi.nlm.nih.gov/geo/query/acc.cgi?acc=GSE205714
Files in database submission	fastq file of K562, Control, H3K4me3, r1, b1, ChIP-seq<br>fastq file of K562, Control, H3K4me3, r2, b1, ChIP-seq<br>fastq file of K562, RBFOX2 KD, H3K4me3, r1, b1, ChIP-seq<br>fastq file of K562, RBFOX2 KD, H3K4me3, r2, b1, ChIP-seq<br>fastq file of K562, Control, Input, r1, b1, ChIP-seq<br>fastq file of K562, Control, Input, r2, b1, ChIP-seq<br>fastq file of K562, RBFOX2 KD, Input, r1, b1, ChIP-seq<br>fastq file of K562, RBFOX2 KD, Input, r2, b1, ChIP-seq<br>fastq file of K562, Control, SUZ12, r1, b2, ChIP-seq<br>fastq file of K562, Control, SUZ12, r2, b2, ChIP-seq<br>fastq file of K562, RBFOX2 KD, SUZ12, r1, b2, ChIP-seq<br>fastq file of K562, RBFOX2 KD, SUZ12, r2, b2, ChIP-seq<br>fastq file of K562, Control, Input, r1, b2, ChIP-seq<br>fastq file of K562, Control, Input, r2, b2, ChIP-seq<br>fastq file of K562, RBFOX2 KD, Input, r1, b2, ChIP-seq<br>fastq file of K562, RBFOX2 KD, Input, r2, b2, ChIP-seq<br>fastq file of K562, Control, H3K27me3, r1, b3, ChIP-seq<br>fastq file of K562, Control, H3K27me3, r2, b3, ChIP-seq<br>fastq file of K562, RBFOX2 KD, H3K27me3, r1, b3, ChIP-seq<br>fastq file of K562, RBFOX2 KD, H3K27me3, r2, b3, ChIP-seq<br>fastq file of K562, Control, Input, r1, b3, ChIP-seq<br>fastq file of K562, Control, Input, r2, b3, ChIP-seq<br>fastq file of K562, RBFOX2 KD, Input, r1, b3, ChIP-seq<br>fastq file of K562, RBFOX2 KD, Input, r2, b3, ChIP-seq<br>fastq file of K562, Control, YTHDC1, r1, b4, ChIP-seq<br>fastq file of K562, Control, YTHDC1, r2, b4, ChIP-seq<br>fastq file of K562, Control, RBM15, r1, b4, ChIP-seq<br>fastq file of K562, Control, RBM15, r2, b4, ChIP-seq<br>fastq file of K562, RBFOX2 KD, RBM15, r1, b4, ChIP-seq<br>fastq file of K562, RBFOX2 KD, RBM15, r2, b4, ChIP-seq

fastq file of K562, Control, Input, r1, b4, ChIP-seq
fastq file of K562, Control, Input, r2, b4, ChIP-seq
fastq file of K562, RBFOX2 KD, Input, r1, b4, ChIP-seq
fastq file of K562, RBFOX2 KD, Input, r2, b4, ChIP-seq
fastq file of K562, siControl, EZH2, r1, b4, ChIP-seq
fastq file of K562, siControl, EZH2, r2, b4, ChIP-seq
fastq file of K562, siControl, SUZ12, r1, b4, ChIP-seq
fastq file of K562, siControl, SUZ12, r2, b4, ChIP-seq
fastq file of K562, siYTHDC1 KD, EZH2, r1, b4, ChIP-seq
fastq file of K562, siYTHDC1 KD, EZH2, r2, b4, ChIP-seq
fastq file of K562, siYTHDC1 KD, SUZ12, r1, b4, ChIP-seq
fastq file of K562, siYTHDC1 KD, SUZ12, r2, b4, ChIP-seq
fastq file of K562, siControl, Input, r1, b4, ChIP-seq
fastq file of K562, siControl, Input, r2, b4, ChIP-seq
fastq file of K562, siYTHDC1 KD, Input, r1, b4, ChIP-seq
fastq file of K562, siYTHDC1 KD, Input, r2, b4, ChIP-seq
fastq file of K562, siControl, Input, r1, b5, ChIP-seq
fastq file of K562, siControl, Input, r2, b5, ChIP-seq
fastq file of K562, siControl, RBFOX2, r1, b5, ChIP-seq
fastq file of K562, siControl, RBFOX2, r2, b5, ChIP-seq
fastq file of K562, siMETTL3 KD, Input, r1, b5, ChIP-seq
fastq file of K562, siMETTL3 KD, Input, r2, b5, ChIP-seq
fastq file of K562, siMETTL3 KD, RBFOX2, r1, b5, ChIP-seq
fastq file of K562, siMETTL3 KD, RBFOX2, r2, b5, ChIP-seq
fastq file of K562, siRBM15 KD, Input, r1, b5, ChIP-seq
fastq file of K562, siRBM15 KD, Input, r2, b5, ChIP-seq
fastq file of K562, siRBM15 KD, RBFOX2, r1, b5, ChIP-seq
fastq file of K562, siRBM15 KD, RBFOX2, r2, b5, ChIP-seq
fastq file of K562, siControl, RBM15, r1, b5, ChIP-seq
fastq file of K562, siControl, RBM15, r2, b5, ChIP-seq
fastq file of K562, siYTHDC1 KD, Input, r1, b5, ChIP-seq
fastq file of K562, siYTHDC1 KD, Input, r2, b5, ChIP-seq
fastq file of K562, siYTHDC1 KD, RBFOX2, r1, b5, ChIP-seq
fastq file of K562, siYTHDC1 KD, RBFOX2, r2, b5, ChIP-seq
fastq file of K562, siYTHDC1 KD, RBM15, r1, b5, ChIP-seq
fastq file of K562, siYTHDC1 KD, RBM15, r2, b5, ChIP-seq
fastq file of K562, WT, Input, r1, b6, ChIP-seq
fastq file of K562, WT, Input, r2, b6, ChIP-seq
fastq file of K562, WT, METTL14, r1, b6, ChIP-seq
fastq file of K562, WT, METTL14, r2, b6, ChIP-seq
fastq file of K562, WT, METTL3, r1, b7, ChIP-seq
fastq file of K562, WT, METTL3, r2, b7, ChIP-seq
fastq file of NB4, RBFOX2 OE, Input, r1, b8, ChIP-seq
fastq file of NB4, RBFOX2 OE, RBFOX2, r1, b8, ChIP-seq

bigWig file of K562, Control, H3K4me3, r1, b1, ChIP-seq
bigWig file of K562, Control, H3K4me3, r2, b1, ChIP-seq
bigWig file of K562, RBFOX2 KD, H3K4me3, r1, b1, ChIP-seq
bigWig file of K562, RBFOX2 KD, H3K4me3, r2, b1, ChIP-seq
bigWig file of K562, Control, Input, r1, b1, ChIP-seq
bigWig file of K562, Control, Input, r2, b1, ChIP-seq
bigWig file of K562, RBFOX2 KD, Input, r1, b1, ChIP-seq
bigWig file of K562, RBFOX2 KD, Input, r2, b1, ChIP-seq
bigWig file of K562, Control, SUZ12, r1, b2, ChIP-seq
bigWig file of K562, Control, SUZ12, r2, b2, ChIP-seq
bigWig file of K562, RBFOX2 KD, SUZ12, r1, b2, ChIP-seq
bigWig file of K562, RBFOX2 KD, SUZ12, r2, b2, ChIP-seq
bigWig file of K562, Control, Input, r1, b2, ChIP-seq
bigWig file of K562, Control, Input, r2, b2, ChIP-seq
bigWig file of K562, RBFOX2 KD, Input, r1, b2, ChIP-seq
bigWig file of K562, RBFOX2 KD, Input, r2, b2, ChIP-seq
bigWig file of K562, Control, H3K27me3, r1, b3, ChIP-seq
bigWig file of K562, Control, H3K27me3, r2, b3, ChIP-seq
bigWig file of K562, RBFOX2 KD, H3K27me3, r1, b3, ChIP-seq
bigWig file of K562, RBFOX2 KD, H3K27me3, r2, b3, ChIP-seq
bigWig file of K562, Control, Input, r1, b3, ChIP-seq
bigWig file of K562, Control, Input, r2, b3, ChIP-seq
bigWig file of K562, RBFOX2 KD, Input, r1, b3, ChIP-seq
bigWig file of K562, RBFOX2 KD, Input, r2, b3, ChIP-seq
bigWig file of K562, Control, YTHDC1, r1, b4, ChIP-seq
bigWig file of K562, Control, YTHDC1, r2, b4, ChIP-seq
bigWig file of K562, Control, RBM15, r1, b4, ChIP-seq
bigWig file of K562, Control, RBM15, r2, b4, ChIP-seq
bigWig file of K562, RBFOX2 KD, RBM15, r1, b4, ChIP-seq
bigWig file of K562, RBFOX2 KD, RBM15, r2, b4, ChIP-seq
bigWig file of K562, Control, Input, r1, b4, ChIP-seq

bigWig file of K562, Control, Input, r2, b4, ChIP-seq
bigWig file of K562, RBFOX2 KD, Input, r1, b4, ChIP-seq
bigWig file of K562, RBFOX2 KD, Input, r2, b4, ChIP-seq
bigWig file of K562, siControl, EZH2, r1, b4, ChIP-seq
bigWig file of K562, siControl, EZH2, r2, b4, ChIP-seq
bigWig file of K562, siControl, SUZ12, r1, b4, ChIP-seq
bigWig file of K562, siControl, SUZ12, r2, b4, ChIP-seq
bigWig file of K562, siYTHDC1 KD, EZH2, r1, b4, ChIP-seq
bigWig file of K562, siYTHDC1 KD, EZH2, r2, b4, ChIP-seq
bigWig file of K562, siYTHDC1 KD, SUZ12, r1, b4, ChIP-seq
bigWig file of K562, siYTHDC1 KD, SUZ12, r2, b4, ChIP-seq
bigWig file of K562, siControl, Input, r1, b4, ChIP-seq
bigWig file of K562, siControl, Input, r2, b4, ChIP-seq
bigWig file of K562, siYTHDC1 KD, Input, r1, b4, ChIP-seq
bigWig file of K562, siYTHDC1 KD, Input, r2, b4, ChIP-seq
bigWig file of K562, siControl, Input, r1, b5, ChIP-seq
bigWig file of K562, siControl, Input, r2, b5, ChIP-seq
bigWig file of K562, siControl, RBFOX2, r1, b5, ChIP-seq
bigWig file of K562, siControl, RBFOX2, r2, b5, ChIP-seq
bigWig file of K562, siMETTL3 KD, Input, r1, b5, ChIP-seq
bigWig file of K562, siMETTL3 KD, Input, r2, b5, ChIP-seq
bigWig file of K562, siMETTL3 KD, RBFOX2, r1, b5, ChIP-seq
bigWig file of K562, siMETTL3 KD, RBFOX2, r2, b5, ChIP-seq
bigWig file of K562, siRBM15 KD, Input, r1, b5, ChIP-seq
bigWig file of K562, siRBM15 KD, Input, r2, b5, ChIP-seq
bigWig file of K562, siRBM15 KD, RBFOX2, r1, b5, ChIP-seq
bigWig file of K562, siRBM15 KD, RBFOX2, r2, b5, ChIP-seq
bigWig file of K562, siControl, RBM15, r1, b5, ChIP-seq
bigWig file of K562, siControl, RBM15, r2, b5, ChIP-seq
bigWig file of K562, siYTHDC1 KD, Input, r1, b5, ChIP-seq
bigWig file of K562, siYTHDC1 KD, Input, r2, b5, ChIP-seq
bigWig file of K562, siYTHDC1 KD, RBFOX2, r1, b5, ChIP-seq
bigWig file of K562, siYTHDC1 KD, RBFOX2, r2, b5, ChIP-seq
bigWig file of K562, siYTHDC1 KD, RBM15, r1, b5, ChIP-seq
bigWig file of K562, siYTHDC1 KD, RBM15, r2, b5, ChIP-seq
bigWig file of K562, WT, Input, r1, b6, ChIP-seq
bigWig file of K562, WT, Input, r2, b6, ChIP-seq
bigWig file of K562, WT, METTL14, r1, b6, ChIP-seq
bigWig file of K562, WT, METTL14, r2, b6, ChIP-seq
bigWig file of K562, WT, METTL3, r1, b7, ChIP-seq
bigWig file of K562, WT, METTL3, r2, b7, ChIP-seq
bigWig file of NB4, RBFOX2 OE, Input, r1, b8, ChIP-seq
bigWig file of NB4, RBFOX2 OE, RBFOX2, r1, b8, ChIP-seq

Genome browser session
(e.g. UCSC)

no longer applicable

Methodology

Replicates

Each sequencing experiment were with two replicates.

Sequencing depth

K562, Control, H3K4me3, r1, b1, ChIP-seq ; 37119667 ; 35890869 ; 121bp ; single-end
K562, Control, H3K4me3, r2, b1, ChIP-seq ; 34142328 ; 33327205 ; 121bp ; single-end
K562, RBFOX2 KD, H3K4me3, r1, b1, ChIP-seq ; 37801427 ; 36588595 ; 121bp ; single-end
K562, RBFOX2 KD, H3K4me3, r2, b1, ChIP-seq ; 34944031 ; 34014554 ; 121bp ; single-end
K562, Control, Input, r1, b1, ChIP-seq ; 5887480 ; 4959227 ; 121bp ; single-end
K562, Control, Input, r2, b1, ChIP-seq ; 8648256 ; 7692745 ; 121bp ; single-end
K562, RBFOX2 KD, Input, r1, b1, ChIP-seq ; 6971243 ; 5888660 ; 121bp ; single-end
K562, RBFOX2 KD, Input, r2, b1, ChIP-seq ; 8544057 ; 7286914 ; 121bp ; single-end
K562, Control, SUZ12, r1, b2, ChIP-seq ; 42488664 ; 41660218 ; 121bp ; single-end
K562, Control, SUZ12, r2, b2, ChIP-seq ; 35683931 ; 33274887 ; 121bp ; single-end
K562, RBFOX2 KD, SUZ12, r1, b2, ChIP-seq ; 27464275 ; 28826168 ; 121bp ; single-end
K562, RBFOX2 KD, SUZ12, r2, b2, ChIP-seq ; 35555679 ; 23951498 ; 121bp ; single-end
K562, Control, Input, r1, b2, ChIP-seq ; 29101890 ; 26758410 ; 121bp ; single-end
K562, Control, Input, r2, b2, ChIP-seq ; 24195690 ; 34956464 ; 121bp ; single-end
K562, RBFOX2 KD, Input, r1, b2, ChIP-seq ; 25933304 ; 25664996 ; 121bp ; single-end
K562, RBFOX2 KD, Input, r2, b2, ChIP-seq ; 27639912 ; 27390507 ; 121bp ; single-end
K562, Control, H3K27me3, r1, b3, ChIP-seq ; 23881346 ; 23576751 ; 121bp ; single-end
K562, Control, H3K27me3, r2, b3, ChIP-seq ; 20173278 ; 19939445 ; 121bp ; single-end
K562, RBFOX2 KD, H3K27me3, r1, b3, ChIP-seq ; 32851239 ; 32473448 ; 121bp ; single-end
K562, RBFOX2 KD, H3K27me3, r2, b3, ChIP-seq ; 26929209 ; 26605131 ; 121bp ; single-end
K562, Control, Input, r1, b3, ChIP-seq ; 17288460 ; 16918980 ; 121bp ; single-end
K562, Control, Input, r2, b3, ChIP-seq ; 21028333 ; 20379447 ; 121bp ; single-end
K562, RBFOX2 KD, Input, r1, b3, ChIP-seq ; 25148103 ; 24495308 ; 121bp ; single-end
K562, RBFOX2 KD, Input, r2, b3, ChIP-seq ; 20758998 ; 20223473 ; 121bp ; single-end
K562, Control, YTHDC1, r1, b4, ChIP-seq ; 46025341 ; 42828791 ; 121bp ; single-end

K562, Control, YTHDC1, r2, b4, ChIP-seq ; 40961547 ; 37857116 ; 121bp ; single-end
K562, Control, RBM15, r1, b4, ChIP-seq ; 41593999 ; 39054952 ; 121bp ; single-end
K562, Control, RBM15, r2, b4, ChIP-seq ; 51469669 ; 47700781 ; 121bp ; single-end
K562, RBFOX2 KD, RBM15, r1, b4, ChIP-seq ; 41730533 ; 39126183 ; 121bp ; single-end
K562, RBFOX2 KD, RBM15, r2, b4, ChIP-seq ; 39956909 ; 37212944 ; 121bp ; single-end
K562, Control, Input, r1, b4, ChIP-seq ; 46964617 ; 46320775 ; 121bp ; single-end
K562, Control, Input, r2, b4, ChIP-seq ; 38684782 ; 38173937 ; 121bp ; single-end
K562, RBFOX2 KD, Input, r1, b4, ChIP-seq ; 42784780 ; 42348929 ; 121bp ; single-end
K562, RBFOX2 KD, Input, r2, b4, ChIP-seq ; 36112804 ; 35715728 ; 121bp ; single-end
K562, siControl, EZH2, r1, b4, ChIP-seq ; 59446318 ; 58151554 ; 121bp ; single-end
K562, siControl, EZH2, r2, b4, ChIP-seq ; 51039350 ; 49962559 ; 121bp ; single-end
K562, siControl, SUZ12, r1, b4, ChIP-seq ; 40221126 ; 39310505 ; 121bp ; single-end
K562, siControl, SUZ12, r2, b4, ChIP-seq ; 40107659 ; 38988864 ; 121bp ; single-end
K562, siYTHDC1 KD, EZH2, r1, b4, ChIP-seq ; 36021950 ; 35221208 ; 121bp ; single-end
K562, siYTHDC1 KD, EZH2, r2, b4, ChIP-seq ; 57269403 ; 55998110 ; 121bp ; single-end
K562, siYTHDC1 KD, SUZ12, r1, b4, ChIP-seq ; 50584003 ; 49307505 ; 121bp ; single-end
K562, siYTHDC1 KD, SUZ12, r2, b4, ChIP-seq ; 36953023 ; 35876269 ; 121bp ; single-end
K562, siControl, Input, r1, b4, ChIP-seq ; 40841348 ; 40365577 ; 121bp ; single-end
K562, siControl, Input, r2, b4, ChIP-seq ; 44270350 ; 43688291 ; 121bp ; single-end
K562, siYTHDC1 KD, Input, r1, b4, ChIP-seq ; 44028779 ; 43570161 ; 121bp ; single-end
K562, siYTHDC1 KD, Input, r2, b4, ChIP-seq ; 37278876 ; 36880855 ; 121bp ; single-end
K562, siControl, Input, r1, b5, ChIP-seq ; 32637379 ; 32128033 ; 80-132bp ; single-end
K562, siControl, Input, r2, b5, ChIP-seq ; 27034271 ; 26571640 ; 80-132bp ; single-end
K562, siControl, RBFOX2, r1, b5, ChIP-seq ; 26922511 ; 22180459 ; 80-132bp ; single-end
K562, siControl, RBFOX2, r2, b5, ChIP-seq ; 26451078 ; 22106491 ; 80-132bp ; single-end
K562, siMETTL3 KD, Input, r1, b5, ChIP-seq ; 62260128 ; 61510620 ; 80-132bp ; single-end
K562, siMETTL3 KD, Input, r2, b5, ChIP-seq ; 32850548 ; 32349561 ; 80-132bp ; single-end
K562, siMETTL3 KD, RBFOX2, r1, b5, ChIP-seq ; 30096051 ; 23790230 ; 80-132bp ; single-end
K562, siMETTL3 KD, RBFOX2, r2, b5, ChIP-seq ; 37907727 ; 29369571 ; 80-132bp ; single-end
K562, siRBM15 KD, Input, r1, b5, ChIP-seq ; 71740918 ; 70891909 ; 80-132bp ; single-end
K562, siRBM15 KD, Input, r2, b5, ChIP-seq ; 59650966 ; 58904328 ; 80-132bp ; single-end
K562, siRBM15 KD, RBFOX2, r1, b5, ChIP-seq ; 24884729 ; 19451216 ; 80-132bp ; single-end
K562, siRBM15 KD, RBFOX2, r2, b5, ChIP-seq ; 29259845 ; 22006354 ; 80-132bp ; single-end
K562, siControl, RBM15, r1, b5, ChIP-seq ; 26287815 ; 23754643 ; 80-132bp ; single-end
K562, siControl, RBM15, r2, b5, ChIP-seq ; 26222506 ; 23547930 ; 80-132bp ; single-end
K562, siYTHDC1 KD, Input, r1, b5, ChIP-seq ; 28112069 ; 27631454 ; 80-132bp ; single-end
K562, siYTHDC1 KD, Input, r2, b5, ChIP-seq ; 60141528 ; 59350379 ; 80-132bp ; single-end
K562, siYTHDC1 KD, RBFOX2, r1, b5, ChIP-seq ; 27516855 ; 21918955 ; 80-132bp ; single-end
K562, siYTHDC1 KD, RBFOX2, r2, b5, ChIP-seq ; 34290272 ; 27132816 ; 80-132bp ; single-end
K562, siYTHDC1 KD, RBM15, r1, b5, ChIP-seq ; 32575846 ; 28506599 ; 80-132bp ; single-end
K562, siYTHDC1 KD, RBM15, r2, b5, ChIP-seq ; 32084743 ; 28267011 ; 80-132bp ; single-end
K562, WT, Input, r1, b6, ChIP-seq ; 29389016 ; 28879999 ; 80-132bp ; single-end
K562, WT, Input, r2, b6, ChIP-seq ; 32344546 ; 31825696 ; 80-132bp ; single-end
K562, WT, METTL14, r1, b6, ChIP-seq ; 18750184 ; 16618783 ; 80-132bp ; single-end
K562, WT, METTL14, r2, b6, ChIP-seq ; 22206633 ; 20115545 ; 80-132bp ; single-end
K562, WT, METTL3, r1, b7, ChIP-seq ; 11162286 ; 10618258 ; 80-132bp ; single-end
K562, WT, METTL3, r2, b7, ChIP-seq ; 12567440 ; 11873800 ; 80-132bp ; single-end
NB4, RBFOX2 OE, Input, r1, b8, ChIP-seq ; 23094095 ; 20453021 ; 80-132bp ; single-end
NB4, RBFOX2 OE, RBFOX2, r1, b8, ChIP-seq ; 47424887 ; 38840282 ; 80-132bp ; single-end

Antibodies

Rabbit polyclonal anti-RBM15; Proteintech; Cat#10587-1-AP
Rabbit polyclonal anti-YTHDC1; Abcam; Cat#ab122340
Rabbit polyclonal anti-EZH2; Cell Signaling Technology; Cat#5246
Rabbit monoclonal anti-SUZ12; Cell Signaling Technology; Cat#3737
Rabbit anti-H3K27me3; Cell Signaling Technology; Cat#9733
Rabbit anti-H3K4me3; Cell Signaling Technology; Cat#9751
Rabbit polyclonal anti-METTL14; Sigma-Aldrich; Cat#HPA038002
Rabbit monoclonal anti-METTL3; Abcam; Cat#ab195352
Rabbit polyclonal anti-RBFOX2; Proteintech; Cat#12498-1-AP

Peak calling parameters

Peaks were called using HOMER in histone (for histone modification ChIP-seq) or factor (for transcriptional factor ChIP-seq) mode with default parameters.

Data quality

Multiple QC checks were performed throughout the analysis including FastQC, reads distribution and duplication rate estimation.

Software

Raw reads were trimmed with Trimmomatic-0.39 (version 0.39)
Trimmed reads were mapped with bowtie2 (version 2.4.1).
Duplicated reads were removed with samtools (version 1.9)
Peaks were called using HOMER (version 4.11)

Flow Cytometry

Plots

Confirm that:

☒ The axis labels state the marker and fluorochrome used (e.g. CD4-FITC).

☒ The axis scales are clearly visible. Include numbers along axes only for bottom left plot of group (a 'group' is an analysis of identical markers).

☒ All plots are contour plots with outliers or pseudocolor plots.

☒ A numerical value for number of cells or percentage (with statistics) is provided.

Methodology

Sample preparation	Cells with different treatments were harvested and washed with chilled PBS, then incubated with anti-human Fc receptor binding inhibitor antibody for 10 min at 4 degree, followed by staining with flow antibodies for 25 minutes at 4 degree in the dark. After washing with FACS buffer once, stained cells were either analyzed immediately or fixed first and detected later
Instrument	BD LSRFortessa or FACSAria III analyzer (BD Biosciences).
Software	Diva (BD Bioscience) software was used to collect data. Flowjo was used to analyze the flow data
Cell population abundance	No cell sorting was applied
Gating strategy	FSC-A/SSC-A was used to gate cell population, then FSC-H/FSC-A was used to gate singlets population and DAPI/FSC-A was used to gate live cells for further analysis.

☒ Tick this box to confirm that a figure exemplifying the gating strategy is provided in the Supplementary Information.
