# Supplementary figures and images for: RBFOX2 recognizes N6-methyladenosine to suppress transcription and block myeloid leukaemia differentiation
Source: Nat Cell Biol. 2023 Aug 28;25(9):1359–68. doi: 10.1038/s41556-023-01213-w (PMC10495261; doi:10.1038/s41556-023-01213-w)

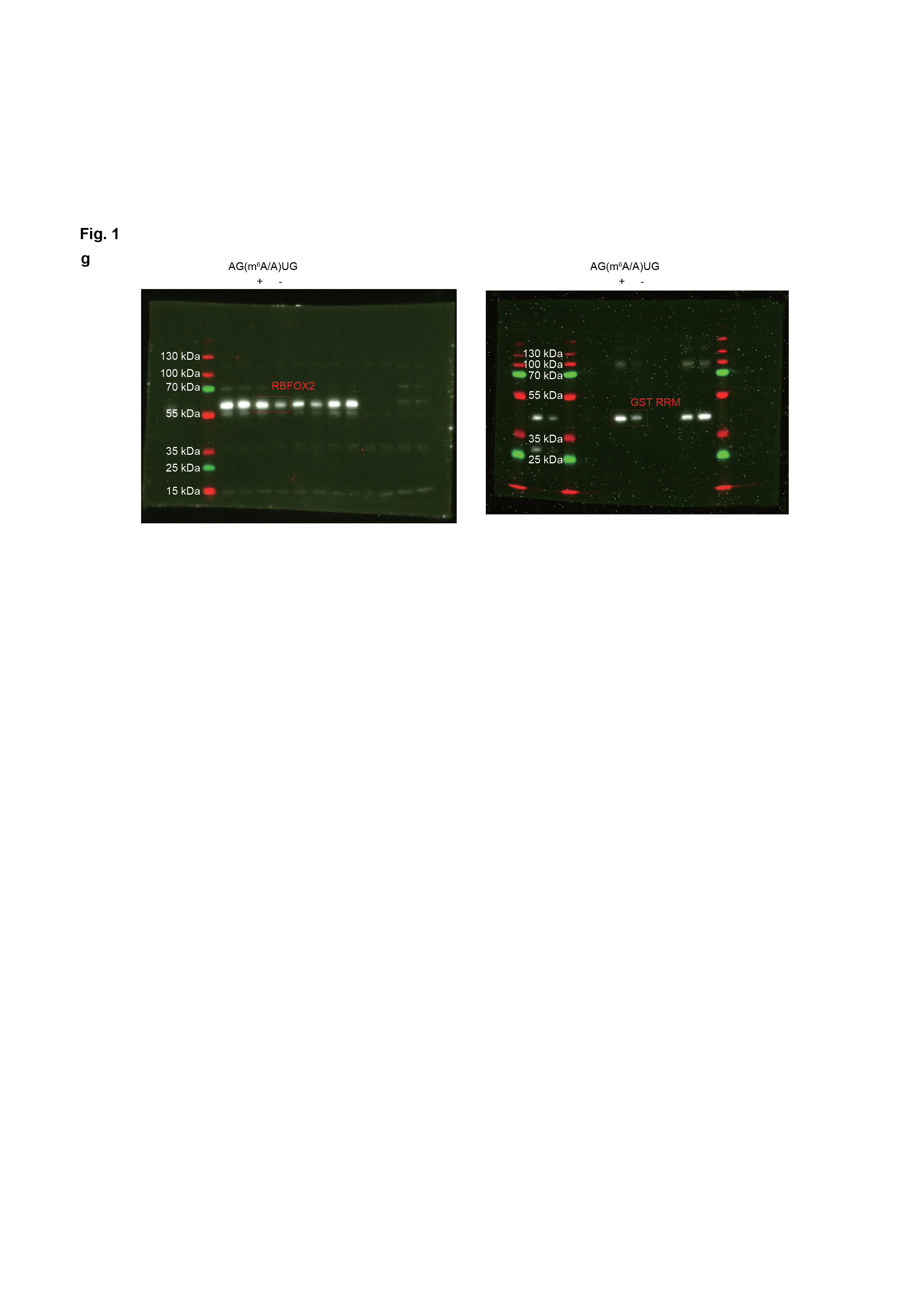

Supplement: Source Data Fig. 1 — Unprocessed western blots. [file 41556_2023_1213_MOESM3_ESM.jpg]

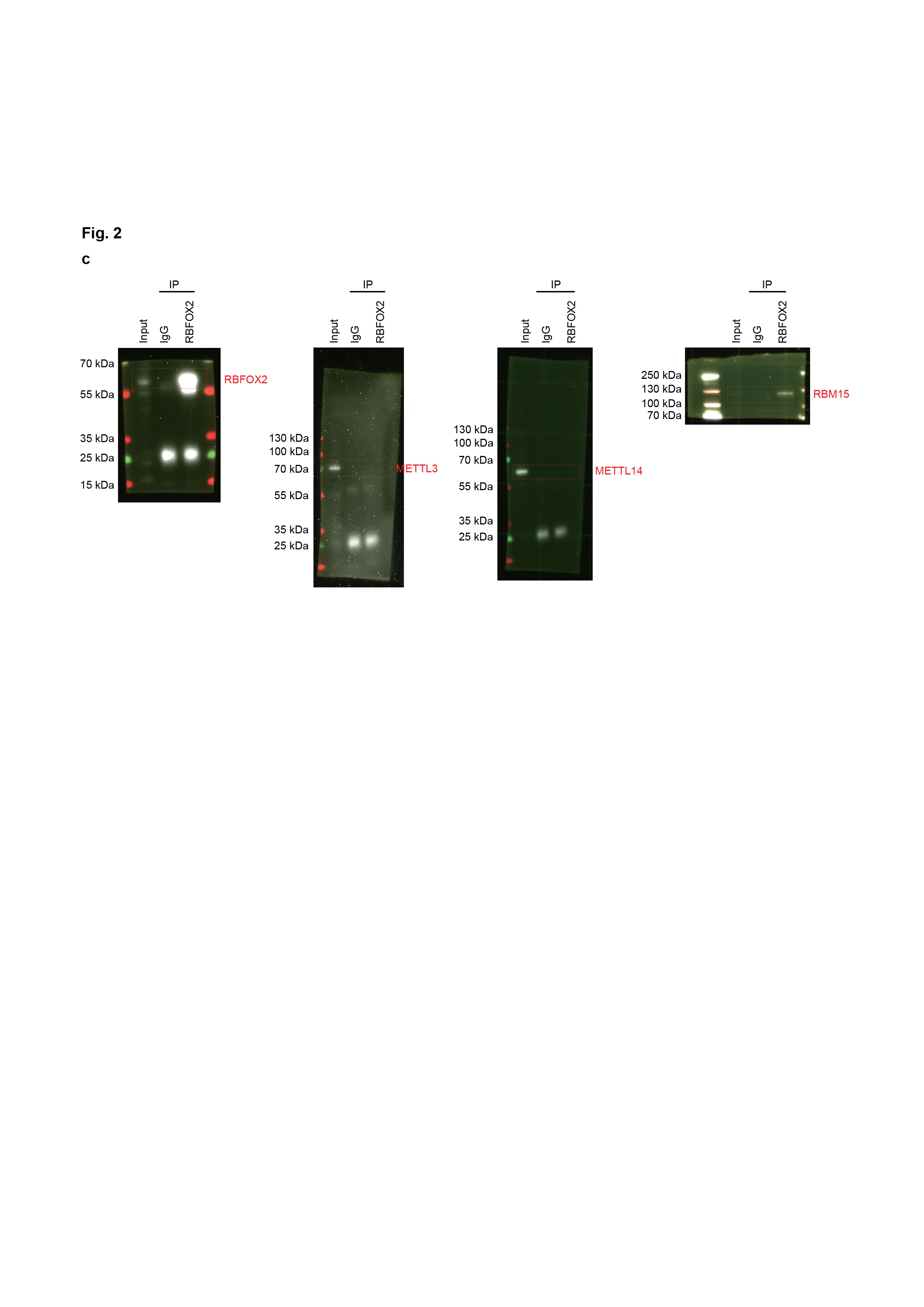

Supplement: Source Data Fig. 2 — Unprocessed western blots. [file 41556_2023_1213_MOESM5_ESM.jpg]

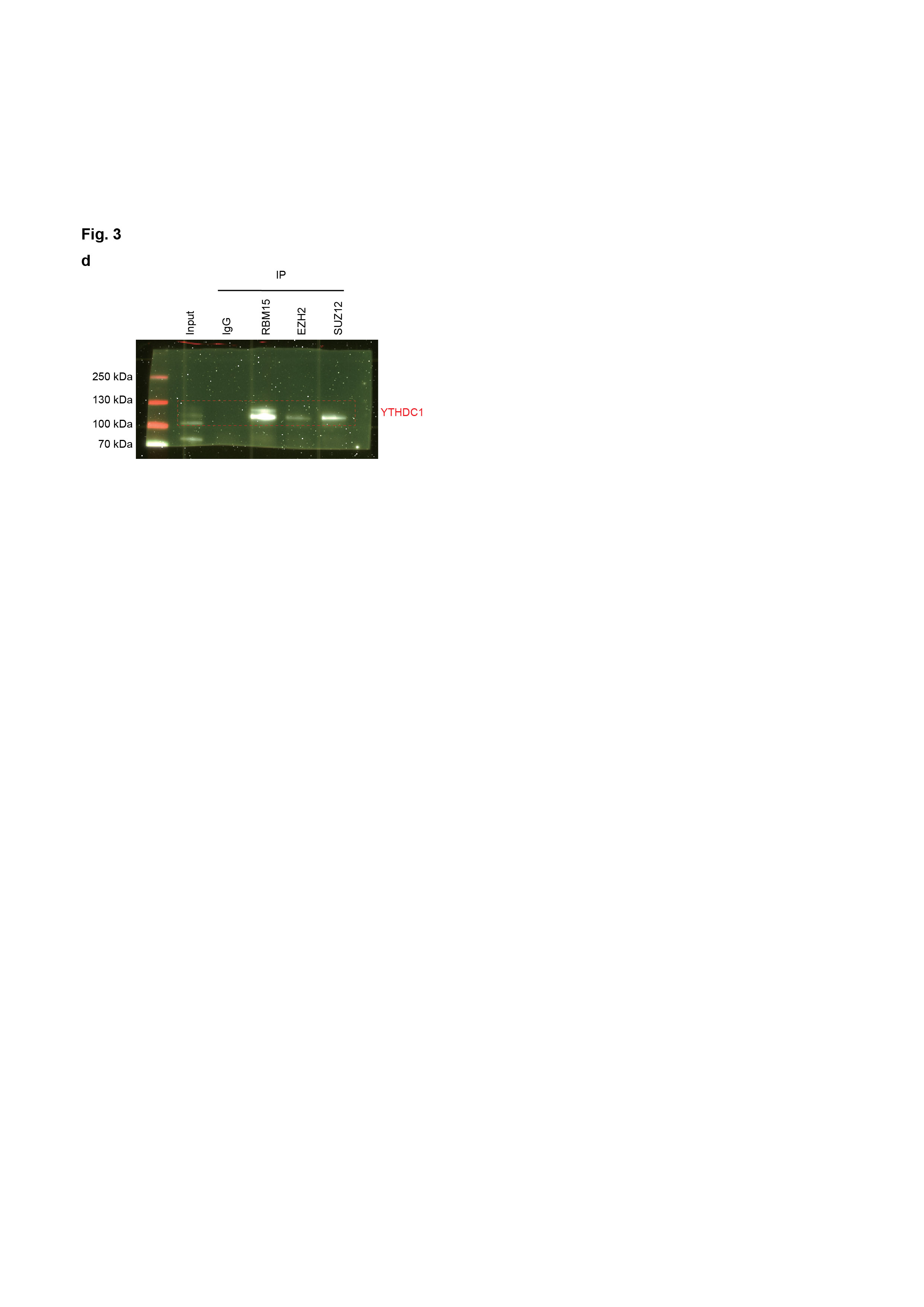

Supplement: Source Data Fig. 3 — Unprocessed western blots. [file 41556_2023_1213_MOESM6_ESM.jpg]

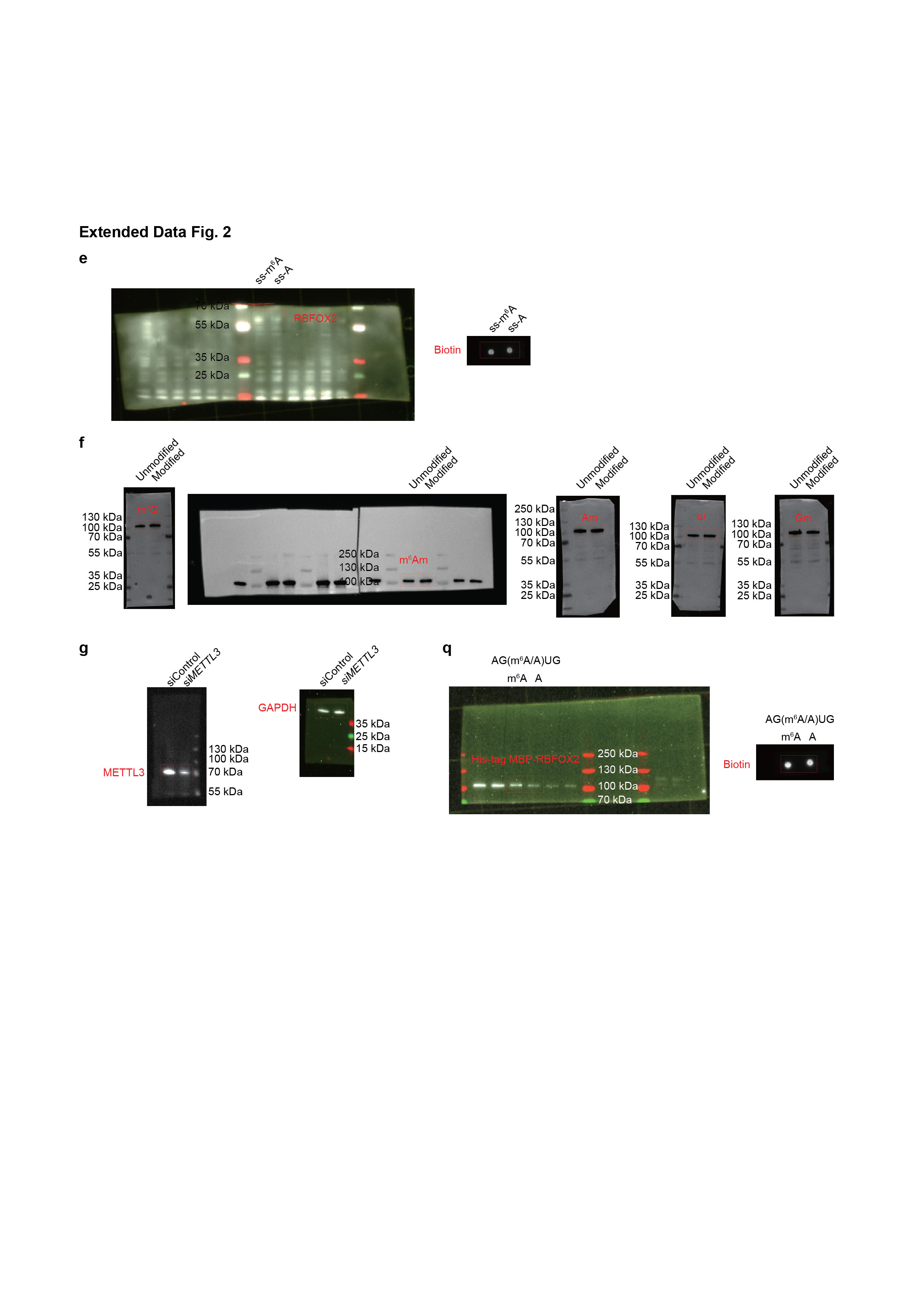

Supplement: Source Data Extended Data Fig. 2 — Unprocessed western blots. [file 41556_2023_1213_MOESM10_ESM.jpg]

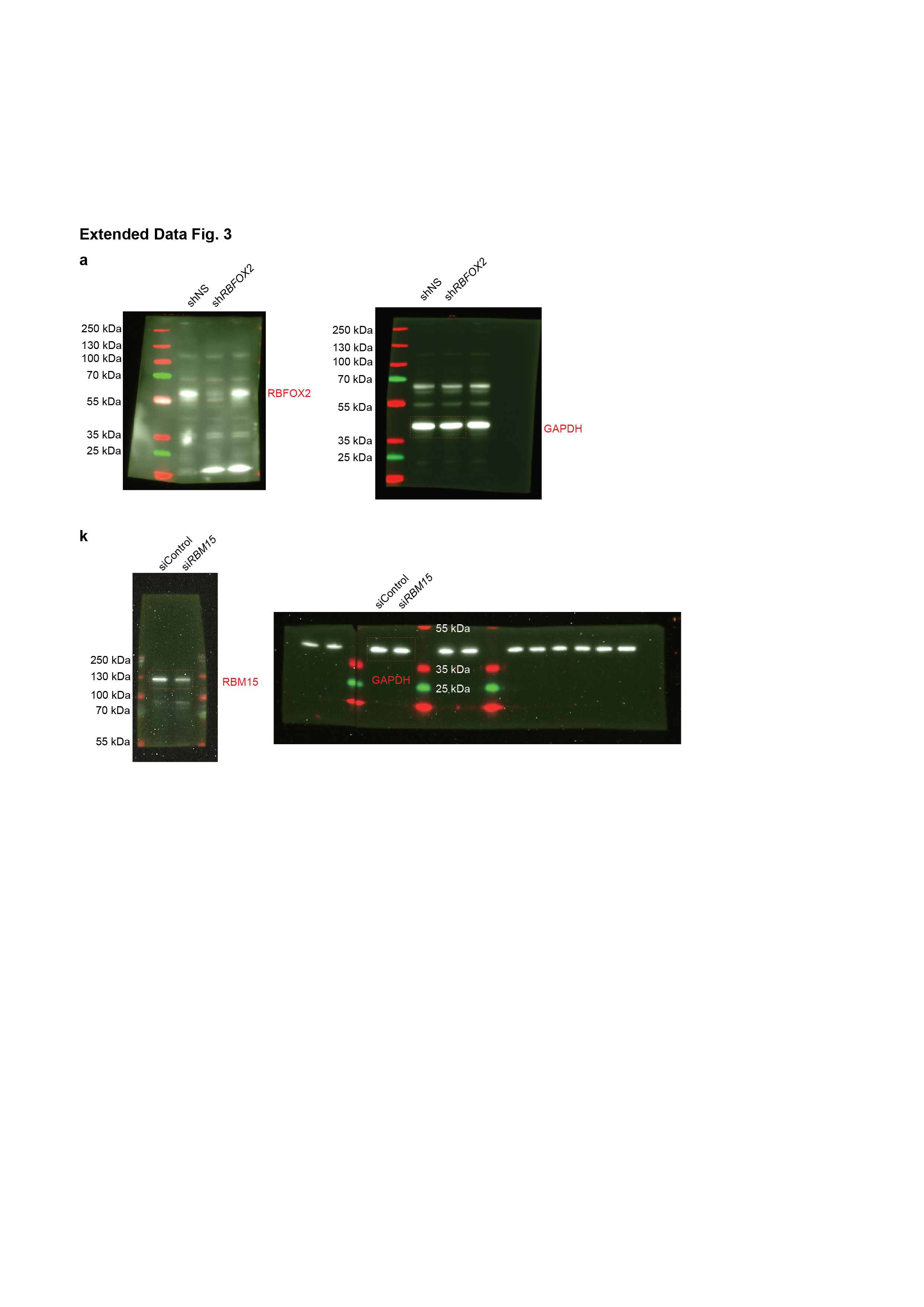

Supplement: Source Data Extended Data Fig. 3 — Unprocessed western blots. [file 41556_2023_1213_MOESM12_ESM.jpg]

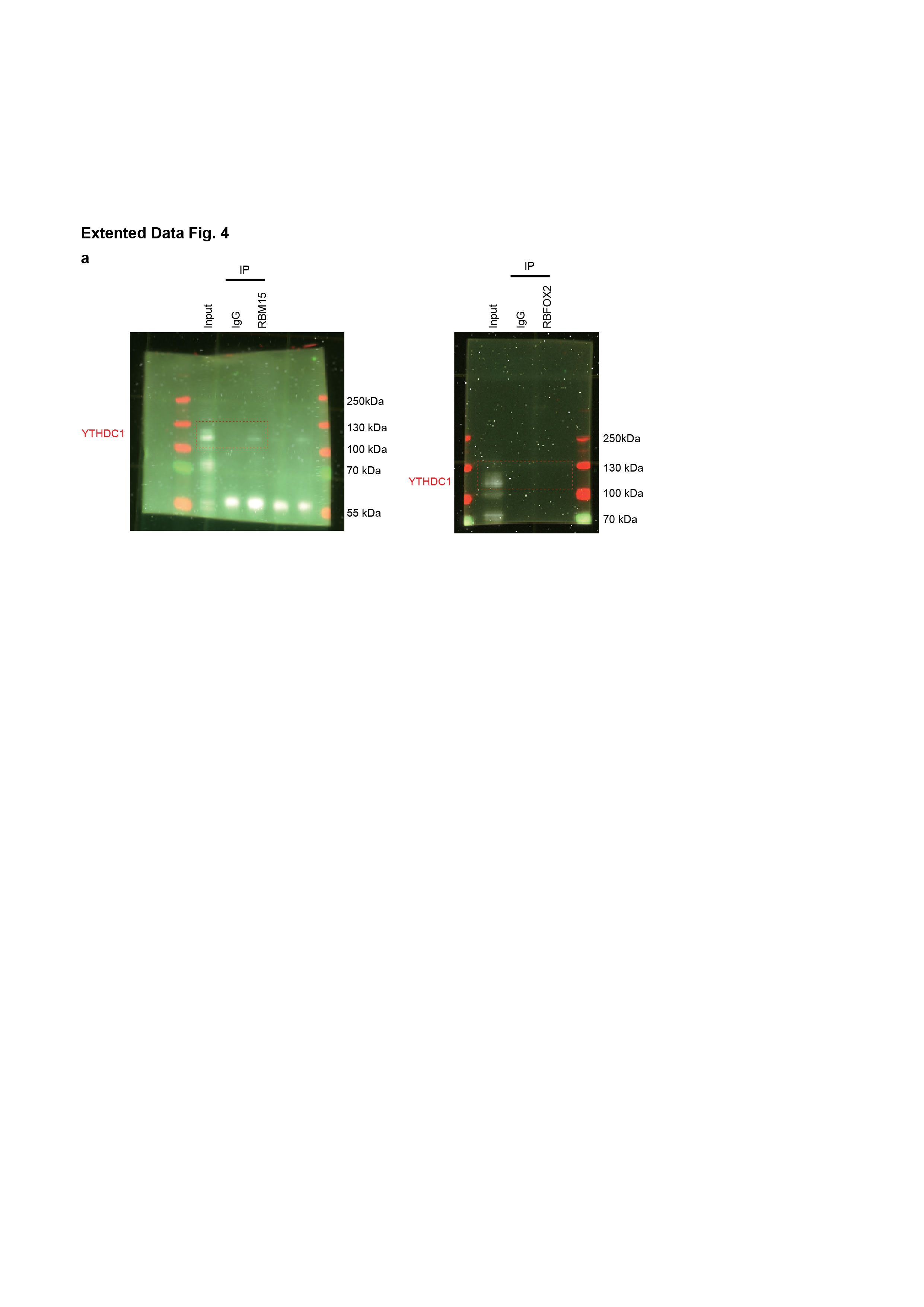

Supplement: Source Data Extended Data Fig. 4 — Unprocessed western blots. [file 41556_2023_1213_MOESM13_ESM.jpg]

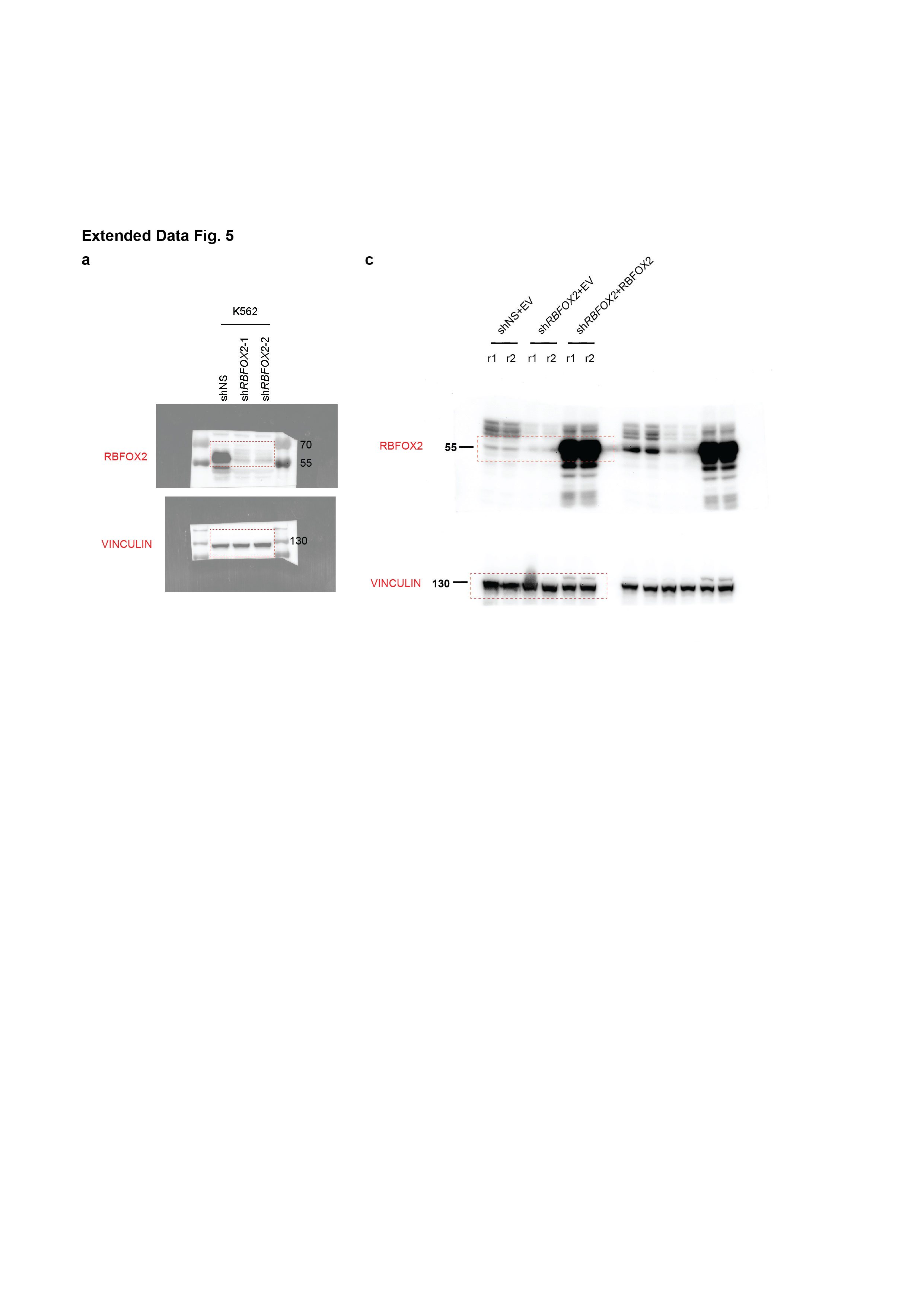

Supplement: Source Data Extended Data Fig. 5 — Unprocessed western blots. [file 41556_2023_1213_MOESM14_ESM.jpg]

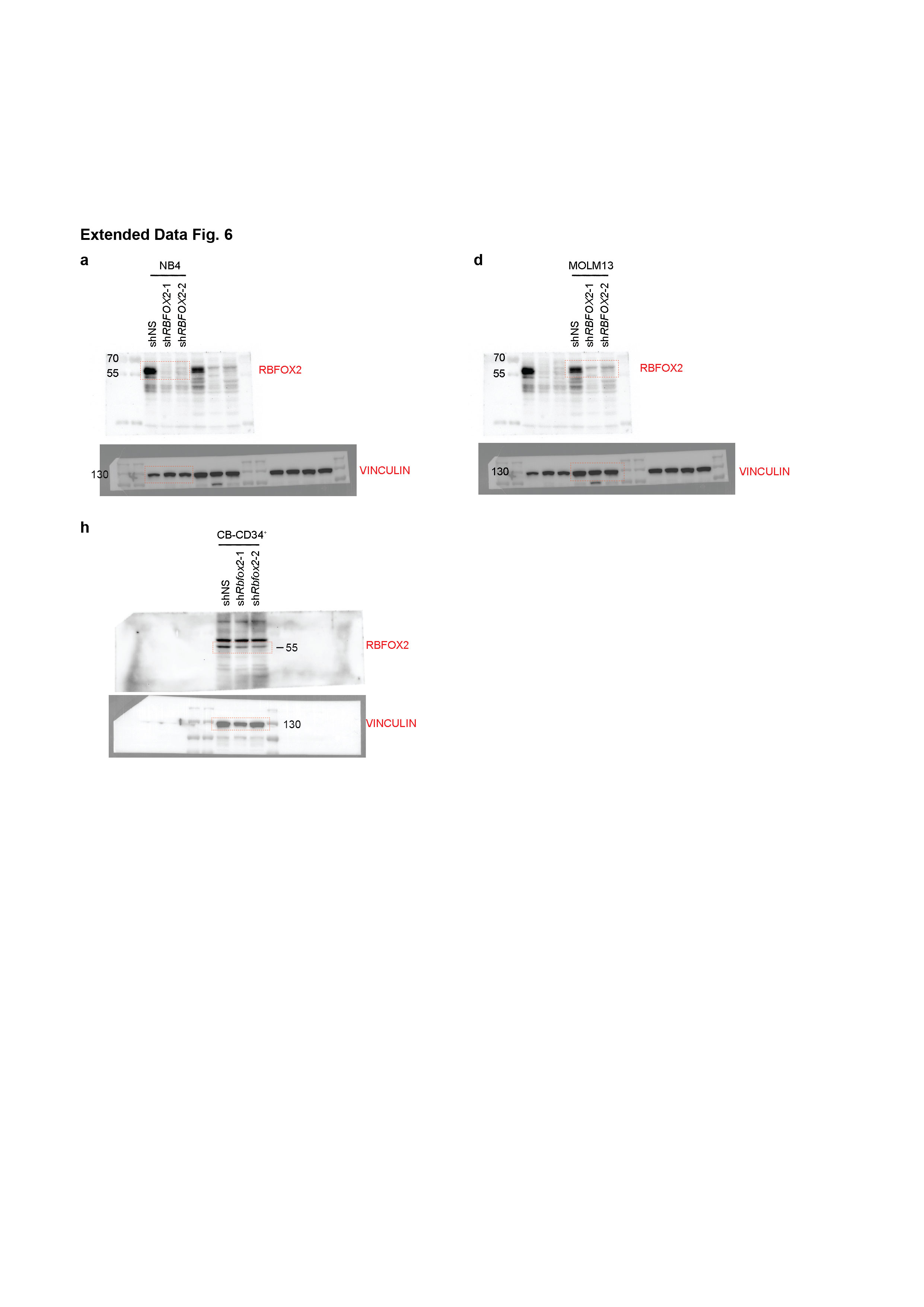

Supplement: Source Data Extended Data Fig. 6 — Unprocessed western blots. [file 41556_2023_1213_MOESM16_ESM.jpg]

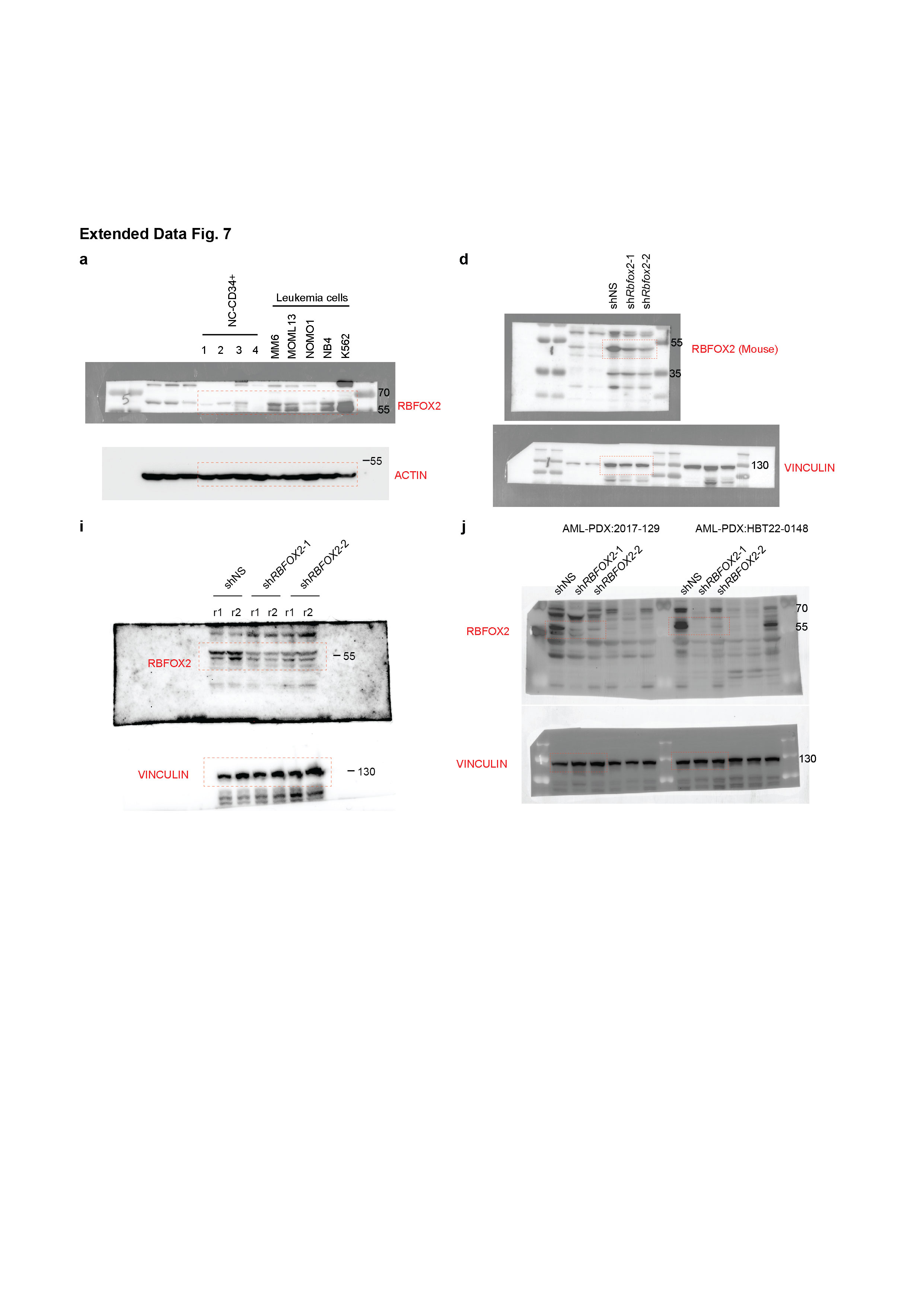

Supplement: Source Data Extended Data Fig. 7 — Unprocessed western blots. [file 41556_2023_1213_MOESM18_ESM.jpg]

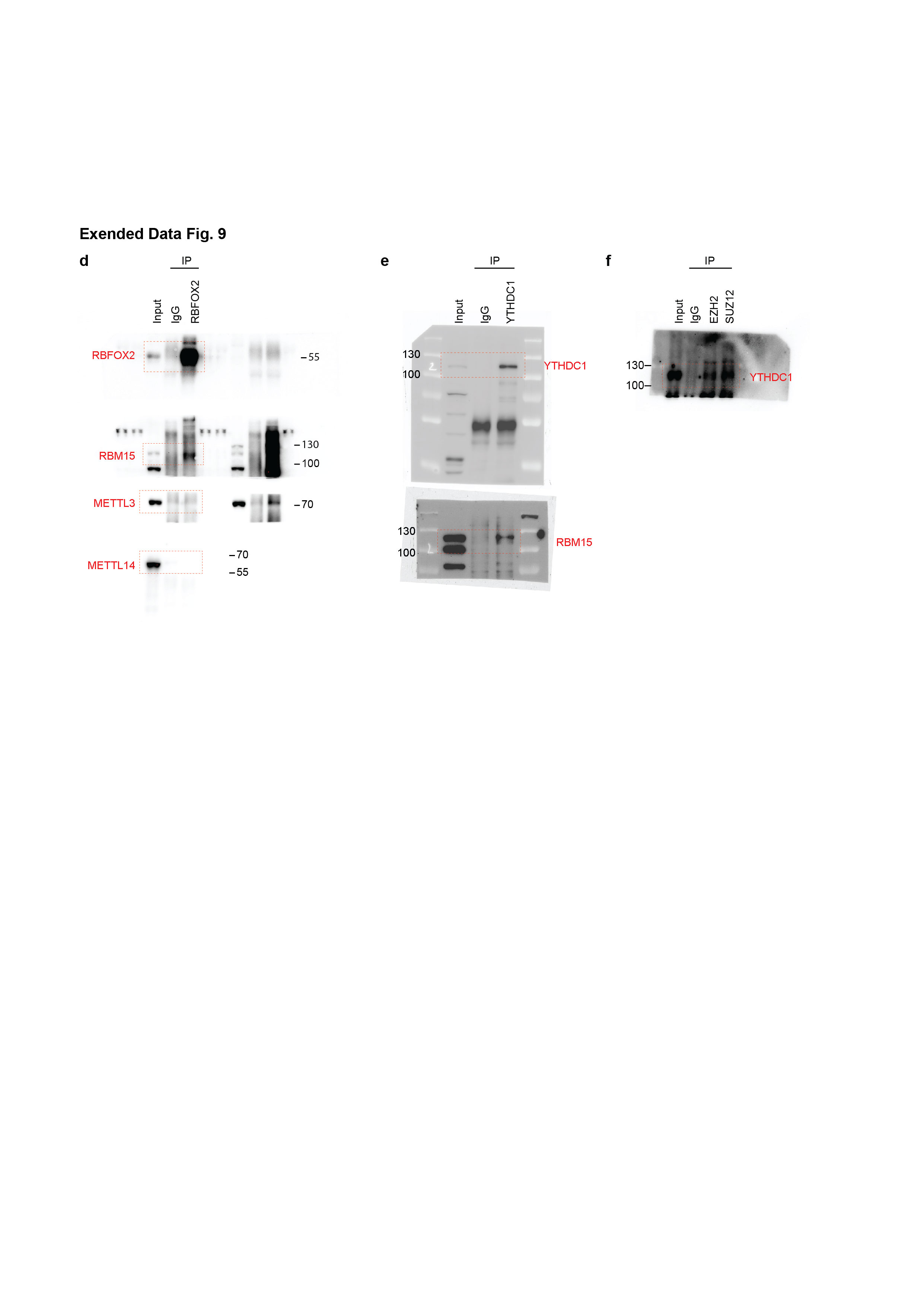

Supplement: Source Data Extended Data Fig. 9 — Unprocessed western blots. [file 41556_2023_1213_MOESM20_ESM.jpg]

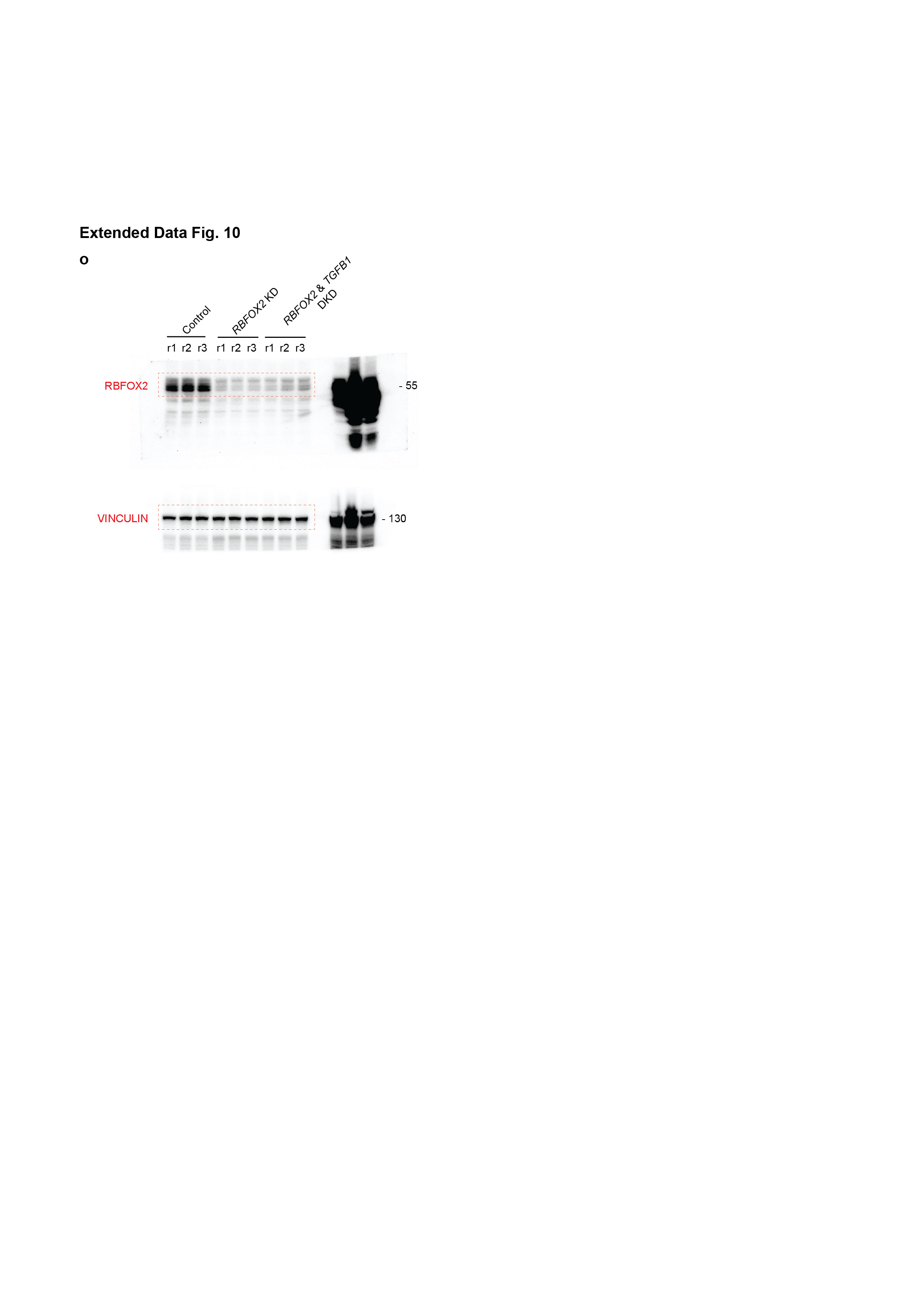

Supplement: Source Data Extended Data Fig. 10 — Unprocessed western blots. [file 41556_2023_1213_MOESM21_ESM.jpg]
